# Face Transplants: An International History

**DOI:** 10.1093/jhmas/jrab019

**Published:** 2021-06-28

**Authors:** Fay Bound Alberti, Victoria Hoyle

**Affiliations:** University of York, UK

**Keywords:** face transplant, surgery, innovation, vascular composite allotransplantation, transplantation, organ donation

## Abstract

Face transplants have attracted global media and public attention since the 1990s. The first recipient, Isabelle Dinoire, found herself at the centre of a dramatic episode of surgical innovation after her transplant was announced in November 2005. Subsequently 47 transplants have been conducted worldwide (including two retransplants) up to August 2020, and these have been accompanied by extensive news coverage. Hundreds of papers on the medical, physical, psychological, and ethical implications of the procedure have been published in the scientific literature, disproportionate to the incidence of the procedure. Face transplants have also featured in films, television, and fiction, indicating an appetite for interrogating the social and interpersonal implications of facial difference. However, the history of facial transplantation has largely been unexplored.

This article provides the first international history of the global development and implementation of facial transplantation. Using published medical literature, media coverage, and oral history interviews with key participants as source material, it situates the experimental transplant in national, institutional, and professional contexts. It argues that charting the history of face transplants over a 30 year period from initial discussions in 1991 to the present provides a valuable case study through which to consider surgical cultures and discourses of medical innovation in the twenty-first century.

## Introduction

Face transplants have been relatively neglected in the histories of medicine and surgery.^1^ This reflects their novelty. The first face transplant occurred in 2005, and there have been no more than 46 around the world. Yet, as a small scale but international phenomenon, face transplants offer a valuable entry point to the history and practice of modern medicine, in particular the evolution and expectations of surgery in the late twentieth and early twenty-first centuries. This includes its geographical, professional, and ethical parameters, the meanings of innovation and the relationship between surgeons and patients, as well as the complex economic, political, and ideological frameworks within which surgical teams work.

Most approaches to face transplants are concerned with the evolution of skill, recorded clinical outcomes, and future research direction, rather than these broad themes, although there is a growing and important body of sociological and philosophical work that sits alongside clinically-informed psycho-social investigation.^2^ Research priorities are themselves a product of medical specialism since the turn of the twentieth century, as funding, clinical, and professional practices have been compartmentalised by distinct disciplinary boundaries. A historiographical, interdisciplinary approach can provide critical new insights to these trends by attending to the ways in which shifting social and cultural contexts shape practices and perspectives over time. An international history not only helps to understand the specific circumstances of the emergence of face transplants in surgical practice, but also offers a case study of how personal and institutional ambition, social structures, and media intervention influence biomedical innovations.

This article is the first to provide an international overview of nearly thirty years of the development and practice of facial transplantation, and it does so primarily through existing documentary sources, supplemented by oral history interviews. It sets out the historical evolution of face transplants, establishing key milestones and themes and focusing on their emergence as an innovation in the treatment of patients with severe facial differences. There remain important questions to be asked of the patient perspective, and the complex philosophical and sociological meanings of the face, but this article specifically engages with the institutional and cultural factors which have enabled an ethically complex and radical surgery to take place. These include opportunity and economic viability, as well as skill, ambition, and understandings of patient need.

## Definitions and Parameters

A face transplant is a type of vascularised composite allograft (VCA), an experimental form of transplantation used primarily in plastic and reconstructive surgery. Aside from face transplants, examples of VCA include hands, upper limb, womb, abdominal wall, and penis. Such transplants involve the transfer of multi-composite tissues and structures, which may include not only skin, but also fat, muscle, nerves, bone, teeth, and hair. Damaged or missing parts of the recipient’s body are replaced, using tissues from a brain-dead donor. In the case of face transplant they can include the face, neck, tongue, and scalp. Although aesthetic outcomes have been the focus of media discussion in face transplants (especially the before-and-after patient image^3^), the primary clinical aim of the transplant is to improve motor, sensory, and communicative functions. In particular, the goals are to enable recipients to eat, speak, blink, and make facial expressions, and enhance quality of life through increased psychological wellbeing and social reintegration. Unlike solid organ transplants, VCA transplants tend not to be considered life-saving (with the exception of the abdominal wall) but life-enhancing. Their visibility sets them apart. With the notable exception of the womb, which has its own set of moral, ethical, and emotional contexts, VCA transplants tend to be visible and can touch or be touched.^4^ As sensorial and emotional organs, they are part of a person’s interface with the social world.

Face transplants, like other transplants, are subject to medical risks. Recipients need a strict regimen of immunosuppressant medications for the rest of their lives, similar to that of solid organ transplant patients, to ensure that the graft does not reject. This medication has well-documented side effects, including an increased risk of infection, cancer, and renal failure. Despite immunosuppression, repeated episodes of rejection are expected. As a result, VCA transplants have long been the subject of ethical debate and, despite technical, immunological, and surgical developments, remain rare. It has only recently been suggested that they be recognised as a standard of care procedure rather than a last resort.^5^

## Methodology and Complexities

It is not straightforward to write an international history of face transplants. Firstly, reliable and substantiated information about surgery rates and outcomes is not always available for political and geopolitical reasons. For example, transplants in China, Russia, and Turkey have taken place but in relatively undocumented circumstances. Secondly, the funding of research in the field by military and other competitive grant-making bodies has led to a lack of data sharing beyond clinical publications. Widespread competition for limited resources and the importance of being original means that some leading surgeons are unwilling to share information or credit. Consequently, as in much scientific research, reports of negative outcomes are less frequent than positive ones. While there are signs of increased cooperation between face transplant programmes in recent years, as opportunities for competitive grants have diminished, most face transplants are conducted in isolation.

Whilst the number of known recipients is low enough to contemplate a complete survey of individual biographies, circumstances, and outcomes, the quality of available information varies significantly across the cohort. Evidence used in this article comes from two key sources: published scientific papers and case reports, and media coverage. In some instances, only brief anonymised reports or online news stories are available. Oral and written testimonies collected from surgical teams, recipients, and other stakeholders supplement these sources. However, the latter are used only where the documentary is lacking; they will form the basis of future publications on specific episodes and aspects of face transplant history.

Some transplant recipients, particularly in the USA, have been widely profiled. In addition to papers in scientific journals about the technical aspects of their surgery, outcomes and prognosis, they have also published memoirs, appeared on talk shows, and been the subject of extensive news coverage. Details about their life circumstances and experiences before and after surgery have been shared, often over many years. However, in a third of cases, recipients have been anonymous, as in Finland, France, Belgium, and Russia, and two examples of transplant in the USA. In these instances, we are entirely dependent on published biomedical information, presented from the point of view of the surgical team. These publications are sparse on details, often in the pursuit of patient anonymity, which makes it difficult to glean more than the barest understanding about the circumstances of the transplant and the biography of the recipient. In other cases, published details about an individual transplant are unavailable and English-language information is only accessible through online news sites.

Additionally, the reporting of facts about transplant recipients, such as age, cause of injury and life circumstance, is often inconsistent. Medical publications and the media do not agree on basic facts, even the date of a transplant. This may be due to the provision of limited information by transplant centres, designed to protect the privacy of the recipient, donor, and their families. However, because news stories appear long before official scientific reports, the proliferation of incorrect information can quickly become widespread. As a result, it is necessary to triangulate from numerous sources to corroborate data, and to balance the veracity of different accounts. This is especially true where details are very personal or considered sensational, for instance whether a traumatic injury might have been self-inflicted. It is therefore possible to make more detailed conclusions on facial transplantation in some regions (e.g., the USA) than in others. To some extent this obscures the multi-national, interconnected nature of the development of facial transplantation, leading to saturation of interest in a handful of high profile cases, as opposed to a global overview.

The cases looked at in this study took place between 2005 and 2020. The first occurred in Amiens, France on 27 November 2005. Three days later photographs of the recipient, a 38-year-old woman named Isabelle Dinoire, made headlines worldwide. The case was widely reported as a medical breakthrough, following years of public speculation about when and where a transplant would take place. Since then a further 46 face transplants have been conducted (up to August 2020 and including two retransplants) in 11 countries, by surgical teams at 21 different hospital and medical facilities (see Supplementary Table 1: Recorded Face Transplants 2005-2020, which sets out the most up-to-date information on all transplants available in the literature). Media interest in the years leading up to 2005 was intense, and fifteen years later the concept and outcomes of face transplantation continue to interest the public, as evidenced by coverage of the face transplant received by 68-year-old Robert Chelsea at Brigham and Women’s Hospital in Boston in 2019, and the retransplant of Carmen Tarleton in July 2020.^6^ Chelsea was the first Black recipient in the USA,^7^ reflecting the lower rates of Black and ethnic minority organ donation in the USA and internationally, and Tarleton was the first person to receive a second retransplant in the USA.^8^ Although this article focuses on the implementation of face transplants between Dinoire’s and Tarleton’s procedures, the surgery was in development for many years before 2005.

## Research in Facial Transplantation, 1991-2005

The Rehabilitation Research & Development Service of the United States Department of Veterans Affairs hosted the first medical conference on Composite Tissue Allotransplantation (CTA), now known as VCA, in Washington, DC in September 1991.^9^ The subject of the meeting was the viability of limb transplantation and it established a long-standing relationship between experimental VCA and the US Department of Defense. It was recognised that military personnel were amongst those most likely to have the injury profiles that required VCA transplantation, such as multiple limb loss and severe facial injury. The discussion was particularly salient in light of the recent Gulf War (August 1990-February 1991). However, at that time it was believed that the high level of immunosuppression required to preserve transplanted skin, which is the most immunogenic of all organs, would be too toxic and dangerous.^10^ Attendees acknowledged that CTA transplantation in humans was at least five years away.^11^

A second CTA symposium was held at the University of Louisville, Kentucky in November 1997, again focused on the potential for limb transplants, specifically hands, although faces were also discussed.^12^ This time the meeting was funded privately, by the Louisville Jewish Hospital Foundation. There are inevitably competing interests in medicine (economic, personal, institutional, nationalistic, ideological, reputational), as in other fields. And it is likely that there was an institutional need to preserve the reputation and distinctiveness of the Kleinert and Kutz Hand Centre, following the retirement of its founder.^13^ An informal survey of the world’s top plastic and reconstructive hand surgeons had suggested that CTA transplantation would be the next significant development in the field, prompting the Hospital to fund research with an “open chequebook.”^14^ The technical, microsurgical skills required were by then well-established and the immunological challenge was the focus of the programme. Soon after the November meeting the research team, led by University of Louisville's John H. Barker, presented data from a study in pigs that showed existing immunosuppressant regimes used in kidney transplants could also be used in limb transplantation. The discovery has since been cited as critical to the expedition of programmes for both hand and face transplant, since all the drugs were already licensed by the Federal Drug Administration (FDA) for use in humans.^15^

Subsequently, the first successful hand transplant was carried out by a team led by Jean-Michel Dubernard and Australian surgeon Earl Owen in Lyon, France on 23 September 1998. This was followed swiftly in January 1999 by a transplant led by Warren C. Breidenbach at Louisville.^16^ Results were mixed. The first recipient, Clint Hallam, was reportedly unable to tolerate the new hand psychologically, and as a result he stopped taking immunosuppressants. Hallam requested the hand’s removal, and an amputation was carried out in the UK 16 months later.^17^ However, the second patient, Matthew Scott, celebrated the seventeenth anniversary of his transplant in 2016, and is reported to have a “just about perfectly functioning” outcome.^18^

The first article on face transplants appeared in the *New Scientist* in October 1998 in the wake of Hallam’s hand transplant.^19^ It posited that hands were the “gateway” to other composite tissue grafts, and speculated that a rivalry between the French team in Lyon and the US team based at Louisville University would spur innovations. Indeed, Dubernard later co-led the team who carried out the first face transplant on Isabelle Dinoire, while Louisville would become a centre in the US development of facial transplantation, establishing a clear developmental link between hand and face VCA programmes.

Although he is unnamed in the article, contextual information makes it possible to identify Louisville’s Barker as the “rival surgeon” who suggested face transplants were the next horizon. A few days earlier Barker had also been quoted in a BBC News article, saying that “a face is just like a hand” and predicting that facial transplantation would quickly follow hand transplants.^20^ The *New Scientist* went on to highlight the practical and ethical issues that would later emerge as the principal debates in the field: the challenge of tissue colour and type matching, the impact of immunosuppression for a non-life-saving operation, the resistance of donor families, the particular emotional resonance of the face, and the question of whether a recipient would look like the donor.

Barker’s analogy between hands and faces was a persistent theme in discussions of face transplants in surgical journals of the early 2000s. The ethicist Françoise Baylis observed this was because a face was “like a hand from the perspective of a surgeon interested in the technical problem of repair.”^21^ Both involve the same parts - muscle, bone, skin, blood vessels, nerves, and arteries – and the same immunosuppressive drug regimens. The risks of rejection are also similar, and they are both life-enhancing procedures with functional benefits. Beyond clinical comparisons, of course, faces and hands are both visible body parts, linked to sexual pleasure, emotional expression, and social communication. These elements (and indeed the psychosocial *differences* between hands and faces) tended to be explored less than their physical commonalities.

Four years after the *New Scientist* article, the British plastic and reconstructive surgeon Peter Butler, based at the Royal Free Hospital London, announced his intention to perform a face transplant at the British Association of Plastic Surgeons (BAPS) winter meeting on 27 November 2002.^22^ This was not the first time face transplants had been proposed by surgeons in the UK or the US. In 1993, *The Observer* reported that Jim Frame, a surgeon in Billericay in Essex, was merely “months away” from carrying out the procedure.^23^ And in 1998, Barker’s team at Louisville spoke publicly of their ambition to undertake the world’s first face transplant.^24^ Butler worked with Frame in Billericay, and his presentation to BAPS built on an article that he published in the *Lancet* in July 2002 with Shehan Hettiaratchy that reported on discussions about the procedure from the 2002 meeting of the Plastic Surgical Research Council in Boston, USA.^25^

These international collaborations were critical to the development of the field, and reveal the often informal context in which innovative ideas take shape. Yet Butler and Hettiaratchy’s 2002 paper seems to have been the first commitment to facial transplantation, as opposed to CTA/VCA in general, in the clinical literature. Hettiaratchy and Butler set out the immunological and surgical challenges of the procedure, particularly the reconnection and regeneration of nerves required to ensure a transplant was functional.

The article did not explicitly engage with psychological concerns (although the authors later would), except to acknowledge that some individuals “have serious physical and psychological problems that cannot be solved by conventional treatments.”^26^ Nor did it address the ethical challenges that would emerge quickly as the idea gained attention. However, in his BAPS paper in November, Butler emphasised that while the surgery was technically feasible it was ethically ambiguous, saying that “I think it is the ethical and moral problems that we face, and that’s where there needs to be full and frank public debate, and that’s where I would like to raise these issues.”^27^

Open discussion about science is an important element of responsible, innovative practice. But striking the right balance among public information, ethical debate, patient wellbeing, and experimental medicine can be challenging. And as the Social Market Foundation, cross-party British thinktank concluded in 2006, the UK media, in particular, sensationalises medical stories, often to the detriment of individuals and organisations concerned.^28^ When the British media latched on to the possibility of a face transplant at the Royal Free, its response was predictably explosive and wildly speculative, culminating in attempts to identify potential recipients.^29^ The tone of the coverage led UK facial difference charity Changing Faces to issue a press release in early 2003 calling upon the Royal College of Surgeons of England (RCS) to investigate the ethics of the procedure before it went further.^30^ A working group was formed, comprised of a panel of medical and psychological experts. The chair of the working group was Professor Peter J. Morris, a specialist in transplant immunology, then President of the RCS; Professor J Andrew Bradley, a professor of surgery at the University of Cambridge, also a specialist in immunology and transplantation; Professor Michael J. Earley, a facial reconstructive surgeon; Mr Martin P Milling, a consultant plastic surgeon specialising in burns injuries, based at Morriston Hospital in Swansea; Professor Len Doyal, a specialist in medical ethics; and Dr Nichola Rumsey, a psychologist who founded and directed the Centre for Appearance Research in Bristol. The group met three times between April and September 2003, and interviewed representatives from leading professional bodies, people living with facial difference, and the surgeons, ethicists, and psychologists on Butler’s team.^31^ In November 2003 they issued a report which echoed Butler’s suggestion that any face transplant “must be proceeded by careful and open debate.”^32^

However, unlike Butler, the working group concluded against proceeding with a face transplant in the UK. Their report set out what would become the main resistance factors, namely:

- The immunological challenges, including the significant potential for acute and chronic rejection, and the side effects and long-term life-shortening impacts of immunosuppression;
- The psychosocial challenges associated with solid organ donation, which would be heightened by the central role of the face in communication, sense of self and identity;
- The paradoxical nature of the ideal transplant patient, who would have to be both desperate enough to require the procedure and resilient enough to withstand its effects;
- The impact on the recipients’ family and the family of the donor;
- The potential for societal misconceptions about people with severe facial differences, in particular that they could not live fulfilling and happy lives.^33^

The question of presumed consent was the most critical pillar of the RCS report’s argument against facial transplantation. The group concluded it would be impossible for a potential transplant recipient to give consent, in light of the lack of evidence and information about risks in comparison to the benefits. They stressed this was not just a matter of surgical and medical risk – indeed, if and when immunological tolerance was achieved, no clinical barriers were anticipated – but the psychological risks. The report suggested that these risks were unknown, and had not been sufficiently discussed. In conclusion, the RCS was “not adverse to facial transplantation,” but stipulated that “until there is further research and the prospect of better control of these complications, it would be unwise to proceed.”^34^

The findings were widely reported as a “moratorium” on face transplants, although the RCS had an advisory rather than a regulatory function.^35^ While the report did not halt the development of a face transplant programme in the UK, it is thought by some to have delayed progress.^36^ The report’s publication in the leading journal *Transplantation* highlighted the case internationally. Shortly afterwards, in February 2004, the National Consultative Ethics Committee for Health and Life Sciences of France (CCNE) released its opinion on a proposal for a programme of five face transplants made by Paris-based surgeon Laurent Lantieri.^37^ Like the RCS working group, the CCNE concluded that there was insufficient evidence of the risks and benefits to proceed ethically. Lantieri had first made public his proposal (as Butler had done) in 2002, when he and his team had begun searching for potential recipients.^38^

Despite their different remits, the RCS and CCNE reports aligned with the dominant thinking of bioethicists, who flagged a number of ethical considerations. As early as January 2003, a short news piece by Arthur Caplan and Dana Katz published in the *Hastings Center Report* argued that the ethical questions facing face transplants were “enormous.” Of particular concern were the effects of immunosuppression, psychological impact, recipient privacy, and donation protocols.^39^ They also highlighted a problem that they felt was downplayed by the surgical teams, namely that where a patient’s previous reconstructions or parts of their surviving tissue had to be removed, the failure of a face transplant would be catastrophic.^40^

By early 2004, four teams were publicly working on developing a face transplant protocol.^41^ These were led, respectively, by Peter Butler at the Royal Free, London; John Barker at Louisville University, Kentucky; Maria Siemionow at the Cleveland Clinic, Ohio; and Laurent Lantieri at Henri-Mondor Hospital, Paris. There was also speculation about the readiness of an unknown Chinese team, who could “easily do it tomorrow” according to RCS working party member Len Doyal, presumably because of less strict ethical protocols.^42^ Overshadowing much of the international debate was media speculation as to which team would be first to perform a transplant in what was dubbed an “international race.”^43^ This language is emotive and sensationalist, but it also reflects a global and ongoing concern about the ways experimental procedures have historically navigated complex (often competing) professional, national, and patient interests.^44^

In contrast to the introduction of new drugs or medical devices, there is no formal regulation of surgical innovation. Instead it is controlled through a myriad of local and national review processes.^45^ Surgeons themselves have a significant role in advocating for and evidencing the value of the experimental procedures they wish to conduct.^46^ Their individual reputations, and the reputations of their institutions, play a part in evidencing the suitability of a new surgical procedure. Personal and professional ambition and curiosity are necessary characteristics of the desire to innovate, and yet those same qualities can lead to charges of egotism – most famously in the case of Christiaan Barnard, who undertook the first successful human heart transplant.^47^ To some extent then, what happens and when is dependent on the ability and willingness of innovative individuals to self-regulate (reflecting the principles of self-regulation upheld by the Royal College of Surgeons of England).

In May 2004, Barker, then Director of Plastic Surgery Research at Louisville University, declared his team’s intention to submit an ethics proposal to the School of Medicine Institutional Review Board (IRB) stating that they were ready to start screening transplant candidates.^48^ Barker argued that patient need drove the decision to perform the procedure, underpinned by attitudinal research into the level of risk that patients were willing to accept. His approach acknowledged the potentially life-shortening impact of a face transplant, but began to identify how that might be justified based on each individual’s right to self-determination.

Barker’s team used analyses of questionnaires taken from people with facial differences, from solid organ recipients, and from the public to suggest that there was a hierarchy of organs in relation to risk. People in all groups were willing to sacrifice more years of their lives to have a new hand than a new foot, or a new larynx than a new hand. Highest of all was the face: people would accept most risk, and the loss of more years of their lives, for the opportunity of a face transplant.^49^

As Ankeny and Kerridge observed, using this study to justify face transplants was based on a conviction that individual patients had the right to decide to have surgery.^50^ The argument conceived of face transplants as like other routine clinical decisions where informed consent was sought. It spoke to the prioritisation of autonomy (one of the four internationally-established principles of medical ethics^51^), and patient choice, implying that to see the situation otherwise – as, for example, in the cases of the RCS and CCNE reports – was paternalistic. It is important to note that cultural differences might have been coming into play here. In the UK context, medicine under the National Health Service (NHS) has traditionally had a less individualist, consumerist approach to medicine (with its connotations of choice and personal/economic investment) than is true of the US context – though that is changing.

Nevertheless, focusing on the importance of individual choice downplayed the experimental nature of the procedure, implying that objective analysis of the risks and benefits could lead to reasonable decisions. According to the RCS and CCNE this would be impossible because the surgery was unproven. At the same time, the responsibility of the surgeon in deciding whether to offer the procedure in the first place was omitted. Any such decision would not be value-free, but rather based on assumptions and beliefs about its efficacy that would be invisible to the transplant recipient. Suggesting a face transplant to a patient might imply that the procedure was a reasonable treatment based on their needs, and that the benefits would outweigh the risks.^52^ These nuances in past constructions of the ethical position are important, because they draw attention to the complex, often invisible power-dynamics at work in relationships between surgeons and patients, and the historical ambivalence of objectivity in any given medical situation.^53^

The Louisville team claimed that they had started actively preparing their face transplant protocol in 2001, and had spent three years refining it for submission, stating that they wanted to ensure it met the strictest ethical criteria.^54^ However, the tensions between preparation and implementation, ethics and surgery, were clear in Barker’s growing impatience with ongoing calls for caution by the summer of 2004: "Tell me what you are going to do during that cautionary period that will get us closer to doing this. Caution by itself will not get us any closer.”^55^ The implication was, as Delaporte has described, that the resistance of ethics committees was “a refusal of the very conditions of knowledge,” in which the absence of evidence or experience meant that evidence or experience could not be gained.^56^

## The Ethics of Facial Transplantation

By this stage in the development of face transplants, the debate was no longer about technical or clinical viability but almost solely focused on the ethical and psychological concerns. The procedure was subject to what Arthur Caplan characterised as “prophylactic ethical debate”: an open and public dialogue about the ethical and social implications of an innovative technology.^57^ This debate took place in both professional and public forums and in full sight of the media. In July 2004, the *American Journal of Bioethics* ran a special issue collating discussions thus far, with contributions from the major international voices in face transplant research, including surgeons, bioethicists, psychologists, and media scholars.^58^

The lead article firmly situated facial transplantation in the history of transplantation more generally, as a field that “has always pushed the boundaries of medicine forward.”^59^ Hand transplants were again characterised as a watershed moment in this history, in shifting the conversation away from the procurement and distribution of organs and towards the balance of risk for patients who received organs not needed to save their lives, but which could have significant positive benefits for their quality of life. While the authors acknowledged that facial transplantation heightened social and identity issues, they were keen to focus on similarities with familiar types of transplantation, and other instances of medical innovation. Though bioethicists stressed the face as a unique case, surgeons pushed for face transplants to be seen on an established continuum of surgical practice. Delaporte highlighted the radical challenge facial transplantation presented to both sides of the argument, requiring a recalibration of ideas about transplantation and plastic and reconstructive surgeries, “between the grave, noble, useful surgery on internal organs and the superficial surgery on surfaces.”^60^ (Importantly, this distinction took place when face transplants were conceptualised *as* surface procedures, whereas many include bone and cartilage as well as facial tissue.)

In working through the bioethical issues, Wiggins, Barker, and Martinez turned to four criteria for ethical medical innovation established by Francis Moore.^61^ These criteria included: 1. Adequate scientific preparation for the innovation; 2. A skilled and experienced team; 3. An ethical climate in the institution wherein the innovation occurs; 4. Open display and public and professional discussion and evaluation before proceeding. The same model had been used to justify human hand transplantation at Louisville.^62^ Concerning the first criterion, the authors repeated the surgical position: that the development of face transplant had reached a point where no more knowledge could be gained from scientific and lab studies. The only way to gain additional knowledge was by performing the procedure. It was impossible to determine whether the harm would outweigh the benefits or vice-a-versa without implementation.^63^ The second criteria, they argued, could be easily assured.

The third criteria was more difficult to meet, as it touched on deeply embedded suspicions that individual egos, institutional reputation building, and potential financial gains were fuelling the face transplant race. Wiggins, Barker, and Martinez attempted to avoid the issue by acknowledging that these motivations were always at play, as institutions and surgeons do trade on their innovations. However, they argued that this was immaterial when all other scientific, surgical, medical, and ethical requirements had been scrutinised and met. Finally, they made a virtue of the public controversies around face transplant, suggesting that public and media debate about the procedure meant it met the requirements of the fourth criterion.

At the same time, Wiggins, Barker, and Martinez argued the psychological risks were similar to those that had been documented in solid organ transplants.^64^ Established protocols could be adapted to meet all of these. Further, drawing on research by the Royal Free team, they stressed psychological effects could be managed and mitigated through careful patient selection and preparation.^65^

Other contributors to the special issue were critical of these arguments, suggesting the Louisville approach failed to account for the fact that a face transplant was a psychological as well as a surgical experiment.^66^ Whilst some understanding of psychological risk was transferable from analogous contexts, many issues were exacerbated in the case of a face transplant. Nichola Rumsey, psychologist and member of the RCS working group, argued that this was because the face was a uniquely significant aspect of identity.^67^ Given what was known about the disruptive impact of facial difference on sense of self, it seemed likely that a new face would be equally, if not more significant. At the very least the impact was unknown. Pressure on the recipient to reintegrate into a normal life, probably under the media spotlight, would also be intense.

Furthermore, the assumptions made about the necessity of face transplant as a treatment with psychosocial benefits were questioned. Whilst surgeons could describe and measure potential functional and aesthetic outcomes, in terms of appearance, eating, speaking, and facial expressions, the promised psychosocial results were unquantifiable. This phenomenon is not restricted to face transplants; modern medicine is founded on quantitative rather than qualitative modes of analysis, and mental health is particularly difficult to quantify. Proponents could only state that because the face is a “predominant anatomical feature” and severe facial disfigurement may cause depression and social isolation, replacing it with a “‘normal’ appearing and functioning face” would have “important psychological benefits.”^68^ The same logic was applied to social benefit: social interactions might be improved by virtue of a more aesthetically attractive appearance that allowed an individual to feel more comfortable in public. What constitutes a positive aesthetic outcome is subjective, and might also include the problematic concept of bodily integrity, the insufficiently theorised idea of physical wholeness that is threatened by physical trauma.^69^

Rumsey suggested these aesthetic arguments made false assumptions about the universally negative experience of severe disfigurement. Research into the psychosocial impact of facial difference showed that severity of disfigurement was not a good indicator of an individual’s level of adjustment or wellbeing. Instead, this was dependent on a variety of other factors including self-esteem, disposition, social skills, and support networks.^70^ Those most likely to experience serious distress because of their appearance were also least likely to cope with the pressures and responsibilities of a face transplant. The search for an ideal patient who was sufficiently needy, robust, and resilient was a paradox. Those most willing to take the risks of a transplant, because of their desperation, were also the most vulnerable to negative psychological effects.

Lessons learnt from the failure of the first hand transplant led to this early focus on patient selection, which foregrounded the importance of both psychosocial readiness and compliance. Samuel Taylor-Alexander highlighted the ways in which face transplant teams constructed a profile of the “ideal patient” to meet these criteria.^71^ This notional individual would have serious functional impairments and psychosocial limitations that justified the risks of the surgery, but would also be psychologically robust enough to manage the after effects. Unlike some of the other surgical teams, Butler stressed that the severity of facial difference alone was not a justification.^72^ In response to the RCS report, Butler worked with psychologist Alex Clarke to take account of studies into the social and individual factors that affect an individual’s coping, and those showing the impact of skills training and coping mechanisms.^73^ They went so far as to suggest blanket ethical approval would not be appropriate, given the highly individualistic factors at work, an approach reflecting surgeons’ interpersonal experiences with individual patients.

The emotional and personal dimensions of the relationship between surgeon and potential transplant recipients was evident. Siemionow based her advocacy for face transplants on personal experience with patients.^74^ Lantieri suggested that only plastic and reconstructive surgeons with direct contact with severely disfigured patients should make determinations on the risk vs benefits of the procedure because “they deal everyday with patients’ distress related to physical and æsthetic disabilities.”^75^ For them, the question as to whether to go ahead with the experimental procedure was not just an ethical one, but also required all involved “to question his/her own soul and conscience.”^76^ The idea that the “soul and conscience” of surgeons were somehow the best determinants of treatment draws attention to the conflicts in medicine as an avowedly dispassionate yet also subjective practice, signalled by the routine invocation of the Hippocratic Oath.^77^

The following year, in 2005, Agich and Siemionow further argued that the ethical debate on face transplants overlooked the experiences and needs of potential recipients. It was “one-sided and sensationalistic,” drawing heavily on film and fiction rather than science and fact.^78^ They suggested that discussions about appearance, psychological risks, and identity transfer obscured the function of the face as an “organ of expressivity.” The purpose of the transplant was to give someone “an organ able to communicate the patient’s feelings and thoughts through facial expression.”

Moreover, they argued, the portrayal of face transplants by bioethicists and the media was akin to the insensitivities of nineteenth-century freak-show audiences. It suggested an inability to accept the reality of living with a severe facial disfigurement: “the opposition to a [face transplant] trial reflects the conviction that people suffering from severe deformity should simply endure their condition; their suffering is apparently not compelling.”^79^ In this view, innovative surgery was a moral duty to the surgeon as well as society, and the weighing of immunosuppression risks against the improvement of quality of life was simplistic, as it did not account for human need. Sociologist Heather Laine Talley has read this position as a symptom of the “disfigurement imaginary,” in which disfigurement is bio-medicalised as a form of ableism, and surgery is justified based on the presumption of the “tragedy of facial differences.”^80^ Delaporte suggests an opposing account, in which the surgical perspective acknowledges that facial difference is not “a disturbance that diminishes the outwards appearance alone” but is central to the relations of human life.^81^

From a historical perspective, these debates about face transplants reveal ongoing and often irreconcilable ideas about the function, limits, promise, and responsibility of surgical innovation.^82^ Opposing camps had clearly emerged prior to the first face transplant. Sharrona Pearl observed that they could be crudely characterised as "surgeons for" and "bioethicists against": the spirit of innovation versus the voices of caution.^83^ As Delaporte argued, this opposition was both inevitable (based as it was on fundamental disagreement about the “nature of the handicap and…the nature of the procedure itself”), and evidence of the limitations of quality of life assessments. After all, “the value of a quality of life lies, above all, *in the living*.”^84^

In 2005, philosophers and other humanities scholars joined surgeons and bioethicists in engaging with the complexities of face transplants. Drawing on the work of the influential sociologist Erving Goffman, they emphasised the significance of the face in the production of identity in society, and called for greater recognition of the social and cultural meanings of the face in the debate.^85^ These meanings were not only relevant to recipients and their families, but also to the families of potential donors. The visibility of the face as opposed to other “deceased donation” organs meant that donor families were more likely to make the connection between the deceased and the recipient, and even want to maintain contact with the face’s new owner.^86^

In failing to engage with the specific ontological status of the face as a social and cultural phenomenon, surgeons and, to some extent, bioethicists, also sidestepped some assumptions about facial difference. Goering pointed out that while efforts to alleviate the distress of severely disfigured patients was admirable, face transplants did not tend to the sources of that suffering.^87^ In other words, the pursuit of face transplants continued to frame the problem of facial difference as an individual deficit when that deficit was societal. Such discussion of social norms inevitably connected the issue of face transplants with concerns about developments in cosmetic surgery and the beauty industry, and the complicity of experimental surgeons in reinforcing damaging cultural practices.^88^

## The First Face Transplant: The Face of Isabelle Dinoire

Ultimately, none of the teams who declared an interest in undertaking the first face transplant did so. Although Maria Siemionow’s team at the Cleveland Clinic in Ohio became the first in the world to receive ethical approval for a face transplant in October 2004 - a success they attributed to a pragmatic science-focused approach – a previously undeclared surgical team in Amiens, France undertook the first procedure.^89^ Benoît Lengelé, One of the surgeons involved suggested it was a conscious strategy for the team to avoid publicising their intentions, preferring to work behind the scenes to ensure the detail was thought through.^90^ Led by maxillofacial surgeon Bernard Devauchelle, assisted by transplantation specialist and member of the French National Assembly Jean-Michel Dubernard (who also undertook the world’s first hand transplant), this team conducted the surgery on 27 November 2005.^91^ The recipient was Isabelle Dinoire, a 38-year-old woman with serious dog bite injuries to her lower face sustained while unconscious from an overdose.^92^ Unlike previous attempts to gain ethical approval, the French team made an application specific to Dinoire’s case, rather than on behalf of hypothetical candidates.^93^ An interdisciplinary team based between Lyon and Amiens supported Dinoire’s psychological and physical recovery, and the surgery was publicised only after the event. In the event, the publicising of the face transplant happened earlier than the team anticipated, due to the operation being leaked to a British tabloid.^94^

It is common for ethical, social, and clinical debates to shift gear once a controversial medical procedure has taken place. After the first face transplant, the conversation shifted away from possible or potential need, which could be subject to debate, and towards the immediate concerns for post-operative care. In the aftermath, the transplant was considered a success from an immunological and functional perspective. Dinoire was able to eat and drink within one week of the surgery, sensation returned quickly to the surface of the skin, and episodes of tissue rejection were successfully managed. Reports on the experience of the patient were minimal, noting only that Dinoire quickly integrated the transplant into her body image and, at 12 weeks post-transplant, was “able to face the outside world and returned progressively to a normal social life.”^95^ When justifying their decision to pursue the operation, the team emphasised the “socially devastating” nature of facial disfigurement, suggesting that it could “lead to depression, social isolation, alcohol abuse and increased risk of suicide in the majority of cases.”^96^ This echoed the recorded perspectives and motives of other leading surgeons.

In a subsequent article on the five year outcomes from the procedure, the surgical team privileged the psychological and social motivations to pursue transplant above the functional challenges that Dinoire had faced in not being able to eat, drink, or speak normally. However, in the results, the aesthetic, functional, and immunological outcomes were emphasised, with only brief reference to the “new life” Dinoire had gained a result of her “new face.”^97^ This was despite the fact that by this time she had undergone treatment for two episodes of acute rejection. Dinoire was also experiencing failing kidney function and receiving treatment for cervical cancer, both of which may have been associated with her immunosuppressant regime. Dinoire’s death from lung cancer at the age of 49, in April 2016, however, was not clinically associated with immunosuppressant use.^98^

The international media response to the news of Dinoire’s transplant was fevered. Initially it focused to the “miracle” of the procedure and awe at the achievements of the surgical team, but this rapidly shifted to interest in the interpersonal histories of Isabelle Dinoire and the donor of her new face.^99^ Pearl has noted an increasingly moralistic tone to the reporting internationally, as the immediate visual impact of the facial transformation wore off.^100^ Although Dinoire’s own voice and perspective were often absent from reporting both immediately and thereafter, speculation about her life, morals, and feelings was widespread.^101^ Less than a week after the surgery, reports emerged that Dinoire had taken an overdose and was unconscious when she was injured.^102^ It discovered that the donor of Dinoire’s face had herself died by suicide, a revelation that cast the relationship between recipient and donor in a new psychosocial light.^103^ Subsequently Dinoire’s actions were scrutinised, including her continued smoking and alcohol consumption, and her “ideal patient” status was questioned.^104^

Nevertheless, the transplant was proof of concept. In the UK, it informed the RCS’s decision to revisit their earlier report and conduct a second investigation into the procedure. Although the opinions of the working party members did not change, and it was felt that evidence of technical and immunological improvements was insubstantial, the inevitability of facial transplantation was accepted.^105^ Acknowledging that further face transplants were likely, including in the UK, the RCS set out a list of pre-conditions essential to a successful programme. However, on 25 October 2006, the day before the report was issued, Peter Butler was granted ethical approval to conduct four face transplants on patients at the Royal Free Hospital in London.^106^

## Research and Development, 2006-2020

The second international face transplant followed quickly after the first, performed in China in April 2006 on a 30-year-old man who was mauled by a bear.^107^ However, the surgery was not rapidly adopted in the way that other experimental transplant programmes have been. In the first year after Barnard performed his now-famous heart transplant (in December 1967), over a 100 further transplants took place, despite controversy and extremely poor prognoses.^108^ More recently, 24 hand transplants took place in the five years after Clint Hallam received his in 1998.^109^ As of 2020 it is estimated that 150 hands have been transplanted worldwide in 45 transplant centres, with around 100 recipients.^110^

**Figure 1.**
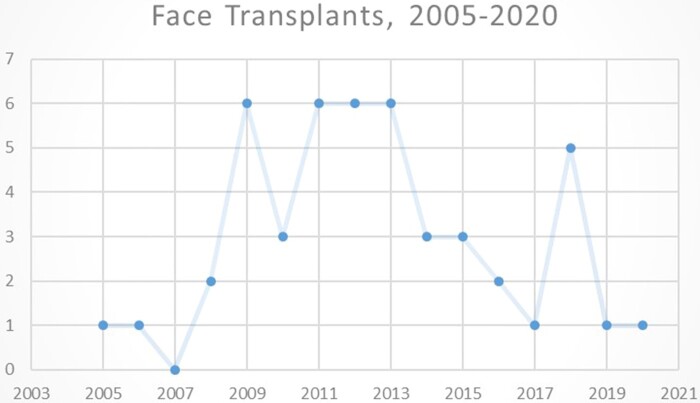
Face Transplants 2005-2020

A smaller number of patients have been involved in face transplants, with only 47 undertaken by the middle of 2020, on 45 recipients.^111^ This may be because there were fewer ideal recipients than anticipated, though donation rates were also low.^112^ Nevertheless, there were emerging patterns to the conduct of surgeries over the implementation period (see Fig 1), which can be understood in relation to economic, social, and cultural factors. No more than six transplants have taken place globally in any one year, with a cluster of 27 transplants occurring between 2009 and 2013. These five years alone accounted for over half of all transplants conducted. They have taken place at 21 hospitals worldwide in 11 countries but only half of those teams have conducted more than one transplant. Indeed, thirty-three of the transplants have taken place in three countries alone: the USA, France and Turkey. Two surgeons account for over a third between them: Bohdan Pomahac at Brigham and Women’s Hospital in Boston (9 transplants, 2009-2019) and Laurent Lantieri at Henri Mondor Hospital in Paris (8 transplants, 2008-2018).

**Figure 2.**
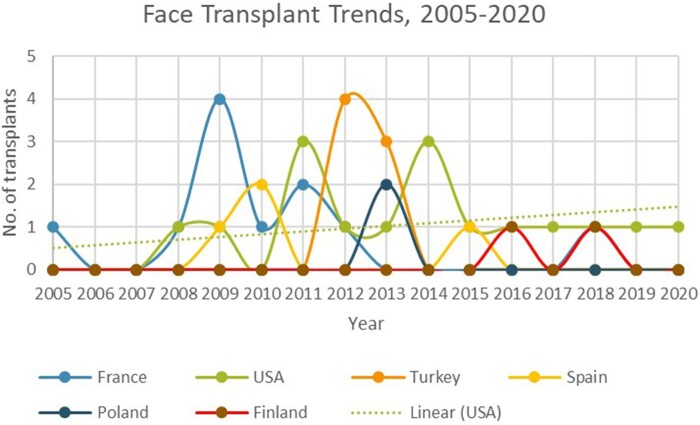
Face Transplant Trends, 2005-2020

The period 2009-2013 appears particularly significant in the implementation period of face transplant. The surgery was conducted only four times between 2005 and 2008, but twenty-seven times in the following five years. It was during this timeframe that the USA, France, and Turkey emerged as world-leaders in the procedure (see Fig. 2 – International Trends, 2005-2020).

In December 2008, Maria Siemionow’s team became the first to perform a transplant in the USA, almost four years after they had received ethical approval. Their recipient, 45-year-old Connie Culp, was a victim of domestic violence, who had been viciously attacked and shot in the face by her ex-husband.^113^ With reference to the importance of incremental developments in innovatice surgery, the transplant was reported as “almost full” as opposed to “partial” - the most extensive and complex to date.^114^ The media response was notably different to the transplants that went before it. Unlike Isabelle Dinoire, Connie Culp was not subject to speculation about her character or the cause of her injuries, and coverage depicted her story as an inspirational journey. In 2009 she appeared on *Oprah* and two years later, in December 2010, became the first face transplant patient to publically meet, thank, and hug the family of her donor.^115^ Connie sadly passed away from an unspecified infection on 29 July 2020.^116^ Yet her active involvement as a patient advocate helped lift the rhetoric of face transplants away from the ethics of surgical innovation to the life-changing and inspirational biographies of recipients.

This latter narrative has persisted most strongly in the USA, and can be seen in the cases of Katie Stubblefield, a 21-year-old woman who became the youngest American recipient in 2017, and Cameron Underwood, a 26-year-old man who received a transplant at NYU Langone in 2018.^117^ Both were survivors of self-inflicted gunshot injuries, formerly a contra-indicator of suitability for transplant for many surgeons, but their journeys of physical, psychological, and emotional recovery have been foregrounded.^118^

The subsequent development of the surgery in the USA was fuelled by the twin drivers of the Department of Defense, on behalf of wounded veterans, and the competitive nature of privatised medicine. Six of the surgeries performed in the key years 2009-2013 took place in the USA, including five by Bohdan Pomahac at Brigham and Women’s Hospital in Boston.^119^ This rapid escalation of activity in the USA can be linked to the interest of the Department of Defense in face transplant as a viable treatment for veterans of the Iraq and Afghanistan wars. A grant of $6.4 million dollars was provided to Brigham and Women’s Hospital in 2009 to support 10 transplants; and the three transplants which have taken place at the Cleveland Clinic were also funded by the DoD.^120^ This level of financing was unavailable in other countries, where a lack of long-term support made it difficult to sustain development. At the same time the need for leading US medical and research centres to differentiate themselves from others for funding reasons has led to strong institutional support for the programmes, while intensifying international interest in the work.^121^

Similar peaks in activity can be observed in France and Turkey. In Paris, Laurent Lantieri, who had previously been refused ethical approval, received funding from the Protocole Hospitalier de Recherche Clinique (PHRC) National to conduct a series of face transplants as clinical research. He subsequently conducted seven transplants between 2008 and 2011.^122^ Unlike in the USA, the majority of recipients were anonymous, with the exception of the first patient, Pascale Coler, and fifth, Jérôme Hamon, who received two transplants and wrote a book about his experiences.^123^ Media coverage was scant. During the same period the team led by Devauchelle and Dubernard conducted a further two transplants at Amiens and Lyon, although almost nothing is publically known about these individuals.^124^ In Turkey a burst of surgeries occurred in 2012 (4 transplants) and 2013 (3 transplants) conducted by three teams. None has been conducted since. Five of the transplants were led by Ömer Özkan at the Akdenzi University School of Medicine, with a recipient profile that was markedly different from elsewhere in the world. Turkish recipients were generally younger (including the youngest ever recipient, Ugur Acar, aged 19) and more likely to have received their facial injury in early childhood.^125^ Patient motivations for transplant were more clearly connected to family and community, with the ability to marry being foregrounded, and subsequent marriages being closely covered by the press.^126^

After 2013, the face transplant landscape changed significantly, with both Turkey and France no longer conducting surgeries (with the exception of the emergency retransplant of Jérôme Hamon in 2018). As a result, the number of operations worldwide declined, demonstrating how limited its adoption had been. The expiry of research funding, and an absence of alternative sources of income, may be the cause. However, Lantieri has subsequently published a survey review of his patients which suggests that the lack of good long-term prognosis was also a limiting factor.^127^ Only in the USA did long term military backing maintain transplant programmes, albeit at a slower pace. Still, on average there has been less than one transplant per year in the USA, with transplant centres going 2-3 years between surgeries. A recent article about the demand for VCA transplantation suggested this low level of activity reflected the very small numbers of people on the national waiting list.^128^ As of December 2017, only two people were on the list, one of whom was probably Robert Chelsea (who had been waiting since 2016 and received his transplant in 2019), and the other Cameron Underwood, who received his transplant in January 2018.^129^

Funding has also been an issue in the development and implementation of facial transplantation elsewhere. In the UK, for example, a face transplant has yet to take place, in part because of difficulties over the use of public healthcare funds. Although Peter Butler’s team at the Royal Free came close to performing the surgery using NHS facilities on at least two occasions, this was only was possible because of the establishment of a charitable foundation to fund the procedure.^130^ A second UK team based in Glasgow was forced to halt their programme when NHS National Services Scotland denied further funding on the basis that too few patients would benefit from it.^131^

It may be the case that the types of injury that justified transplant elsewhere, specifically facial gunshot wounds, severe electrical burns, and industrial accidents have been less common amongst the UK population due to gun control laws and health and safety regulations. However, this did not entirely explain the absence of a transplant, when small numbers of potential recipients have not stopped the procedure elsewhere. Leaving aside any outstanding ethical considerations, whether or not a transplant will ever occur in the UK depends on whether an NHS Trust is willing to risk investing the significant sum of money required despite the potential for failure and the long-term patient aftercare. The authors are currently writing an article specifically about the UK experience, which will illuminate these national complexities further.

Where face transplants have recently occurred elsewhere, funding has been by public healthcare, entirely or in part. In a small number of cases, health insurance providers have contributed, marking a potentially important shift in how far the life-enhancing potential of face transplants is a “manifestly ethical goal.”^132^ The transplant of Maurice Desjardins in Canada in 2018, for example, was funded by the public health system, while a third of the cost of Cameron Underwood’s transplant in New York in 2018 was provided by his insurance company (the remainder came from a Department of Defence grant).^133^ The newest face transplant programme, at Helsinki University in Finland, has been funded entirely under the public healthcare system.^134^

Recent ethical contributions to the debate suggest that the life-shortening nature of the drugs required do not, in themselves, count against the procedure. Quantity of life is not more important than quality of life, and therefore a transplant may still be considered the best standard of care available.^135^ These developments are critical to the future of face transplants, for they help determine their establishment as a standard of care rather than as time-limited clinical research. Other determinants include the development of longitudinal and comparative data (the latter of which is difficult when some individuals and organisations remain averse to data sharing), and how success has been evaluated. Some continued questions over the future of face transplants include the ability to incorporate psychological wellbeing into evaluating outcomes and the extent to which alternative, equally innovative procedures such as tissue engineering might come to the fore.^136^

## Conclusion

An international history of facial transplantation, as an innovative form of surgery, provides insights into how this practice has occurred in different biomedical, ethical, economic, cultural, and social contexts. And it reveals significant features. In the first instance, military funding prompted programmes of work and research and an influx of potential patients in the USA, caused by the Gulf War in the early 1990s, and exacerbated by the conflicts in Afghanistan and Iraq in the 2000s. The types of injuries received in combat – such as gunshot wounds or explosive impact injuries – are those that lend themselves to reconstruction via VCA transplants of hands and faces. Significant investment of funds by the Department of Defense in American innovation led to a surge in face transplants from 2008 onwards, which was then matched in other parts of the world. The effect was further amplified by the privatised, competitive medical sector, which led institutions to support high risk, long-term programmes of treatment in order to build national and international reputations. In the process a small number of high-impact surgeons, who have been building face transplants into their professional portfolios, tend to be the focus of media discussion.

This explains, to some extent, the persistent rhetoric of competition and rivalry that has characterised the development of VCA, and the media framing of a face race. It is true that surgeons, institutions, and countries accrue kudos and credibility by being first to accomplish an innovative treatment. But that is not to say that innovation cannot also be an appropriate therapeutic intervention. The history of scientific medicine is, after all, a history of experimentation, with tangled political, economic, personal, and professional motivations.^137^ Yet innovative surgery attracts a different level of media and public scrutiny to drugs and medical devices. The Royal College of Surgeons of England’s working party, the “prophylactic ethical debate,” and the media speculation around face transplant became influential in determining whether or not the treatment was viable. In risk averse environments, such as the UK, what has been depicted as widespread scrutiny in the court of public opinion may have inhibited innovation, despite the readiness to proceed.^138^ So the respective roles of media and public opinion need to be included in the institutional, professional, emotional, economic, and international contexts.

Investigating the history of facial transplantation may help to understand its impact on health and wellbeing, its future as a procedure, and the contexts in which it takes place. This includes the complex ways that ethics, skill, need, innovation, and opportunity have been defined and negotiated. Defining patient benefits via ethical and psychological protocols are necessarily central to discussions, though debate can be polarised. And despite the uniqueness of face transplants as a procedure, there is much to be learned from other fields. Assessing patient-reported quality of life outcomes, for instance, are relatively novel in face transplants, but core practice in other forms of facial surgery.^139^

It is therefore encouraging that some leading surgical teams have been building on multi- and interdisciplinary collaborations to address critical issues about psychological impact and attempting to find ways of measuring emotional health of patients. This is important in helping to redefine successful outcomes in historically-informed and culturally sensitive ways.^140^ As life-enhancing transplantation moves towards becoming a standard of care, ways to measure these outcomes become essential for building ethical frameworks. More nuanced ethical debates are emerging, which acknowledge the necessity of transparency of outcomes, address competition and conflicts of interest, and recognise the psychosocial impact of the procedure on recipients, their families, and donor families.^141^ These are ongoing questions about the longitudinal impact and social meanings of face transplants which require collaborations between the arts and the sciences, and across global teams.

Yet practical and financial issues remain. As defence funding comes to an end in the USA, there is as yet no published evidence of military usage of the procedure. This may be accounted for by the range of challenges that the surgery still faces, with reference to ethics, the measurement of outcomes, poor long-term prognosis for recipients, and the difficulty of finding donors.^142^ Without financial backing, the continuation of facial transplantation will have to become a standard of care and covered, at least in part, by medical insurance or public health care. Otherwise, instances of the procedure may decline further, until face transplants are overtaken by other innovative treatments that are some years away, such as tissue regeneration. In this case the history of face transplants may be a short one.

## Supplementary Material

See Supplementary Table 1, Supplementary Material online.

## Supplementary Material

jrab019_Supplementary_Data

## Footnotes

1 This is the case in general histories as well as more specific discussions of plastic and reconstructive or aesthetic surgery. Thomas Schlich, ed., *The Palgrave Handbook of the History of Surgery* (London: Palgrave Macmillan, 2018); Paolo Santoni-Rugiu and Philip J. Sykes, *A History of Plastic Surgery* (New York: Springer, 2007); Yong Bae Kim, “The History and Future of Plastic and Reconstructive Surgery,” *Archives of Plastic Surgery* 42 (2015): 515. More recent cultural investigations include Fay Bound Alberti, “From Face/Off to the Face Race: The Case of Isabelle Dinoire and the Future of the Face Transplant,” *Medical Humanities* 43 (2017): 148–154; Sharrona Pearl, *Face/On: Face Transplants and the Ethics of the Other* (Chicago: University of Chicago Press, 2017); Suzannah Biernoff, “Theatres of Surgery: The Cultural Pre-history of the Face Transplant,” *Wellcome Open Research* 3 (2018).

2 Heather Laine Talley, *Saving Face: Disfigurement and the Politics of Appearance* (New York: New York University Press, 2014), 29-30; Marie Le Clainche-Piel, "Greffe de visage, regard sociologique [A sociological perspective on facial transplant]," *Soins*. 839 (2019): 33-35; Anne Marie Martindale and Pamela Fisher, "Disrupted Faces, Disrupted Identities? Embodiment, Life Stories and Acquired Facial ‘Disfigurement’," *Sociology of Health & Illness* 41 (2019): 1503-1519; François Delaporte, *Figures of Medicine: Blood, Face Transplants, Parasites* (New York: Fordham University Press, 2013).

3 Fay Bound Alberti, "Face Transplants as Surgical Acts and Psychosocial Processes," *Lancet* 395 (2020): 1106-1107.

4 E.D. Carosella & T. Pradeu, “Transplantation and Identity: A Dangerous Split?” *Lancet* 368; 9531 (2006): 183-184. Abdominal wall transplants are not visible in the same way as hands and faces, but neither do they carry the same cultural meanings as the womb.

5 A. L. Caplan, B. Parent, J. Kahn, W. Dean, L.L. Kimberly, W.P.A. Lee, and E.D. Rodriguez, “Emerging Ethical Challenges Raised by the Evolution of Vascularized Composite Allotransplantation,” *Transplantation* 103 (2019): 1240–1246.

6 J. Ducharme, “Meet the First African-American Face Transplant Recipient,” *Time*, October 2019, https://time.com/5709294/first-african-american-face-transplant/.

7 Pascale Coler was the first Black face transplant recipient internationally. See C.J. Vercler, “Ethical Issues in Face Transplantation,” *AMA Journal of Ethics* 12 (2010): 378-382.

8 NHS Blood and Transplant, "Organ Donation and Transplantation Data for Black, Asian and Minority Ethnic (BAME) Communities, Report for 2017/2019," (2019), https://nhsbtdbe.blob.core.windows.net/umbraco-assets-corp/12048/bame-organ-donation-and-transplantation-data-2017-18.pdf; Michael Case, “New Hampshire Woman Becomes First Person to Get Second Face Transplant in U.S.,” *Time*, 7 August 2020, https://time.com/5877653/carmen-blandin-tarleton-second-face-transplant/.

9 John H. Barker, Warren C. Breidenbach, and Charles W. Hewitt, “Introduction: Second International Symposium on Composite Tissue Allotransplantation held at Jewish Hospital in Louisville, Kentucky, May 17-18, 2000,” *Microsurgery* 20 (2000): 357.

10 R. Llull, “An Open Proposal for Clinical Composite Tissue Allotransplantation,” *Transplantation Proceedings* 30 (1998): 2692–2696.

11 Barker, Breidenbach, and Hewitt, “Introduction."

12 The conference led to a special issue of *Transplantation Proceedings* (Vol 30, Issue 6, 1998), edited by John H. Barker, Jon W. Jones, and Warren C. Breidenbach.

13 Interview with John H. Barker MD, formerly Director, Plastic Surgery, University of Louisville and Scientific Director, Jewish Hospital Foundation; currently Friedrichsheim Professor of Experimental Orthopedics and Trauma Surgery, JW Goethe University, Frankfurt, Germany, 2 April 2020.

14 Ibid.

15 Ibid.

16 Jean-Michel Dubernard, E. Owen, G. Herzberg, M. Lanzetta, X. Martin, H. Kapila, M. Dawahra, N. Hakim, “Human Hand Allograft: Report on First 6 Months,” *Lancet* 353; 9161 (1999): 1315-1320.

17 Lawrence K. Altman, “A Short, Speckled History of a Transplanted Hand,” *New York Times*, 27 February 2001, https://www.nytimes.com/2001/02/27/science/the-doctor-s-world-a-short-speckled-history-of-a-transplanted-hand.html; J. Slatman and G. Widdershoven, "Hand Transplants and Bodily Integrity," *Body & Society* 16 (2010): 69-92.

18 “Second Hand Transplant Recipient Reaches 15 Year Milestone,” *Jewish Community of Louisville*, 28 April 2016, https://jewishlouisville.org/second-hand-transplant-recipient-reaches-15-year-milestone/; personal communication Professor Bohdan Pomahac, Brigham and Women’s Hospital, Boston, 9 June 2020.

19 “In Your Face,” *New Scientist*, 3 October 1998, https://www.newscientist.com/article/mg16021540-100-in-your-face/.

20 BBC News, “From Hand to Face,” 30 September 1998, http://news.bbc.co.uk/1/hi/health/183870.stm.

21 Françoise Baylis, “A Face is Not Just Like a Hand: Pace Barker,” *American Journal of Bioethics* 4 (2004): 30-32, 30.

22 Natasha McDowell, “Surgeons Struggle with Ethical Nightmare of Face Transplants,” *Nature* 420**; **449 (2002), https://doi.org/10.1038/420449a.

23 M. Halle, "Face Transplant Offers Hope to Disfigured," *The Observer*, 10 October 1993, 6; interview with Jim Frame, 20 May 2020.

24 Stuart Miller, "Face Transplants a Step Nearer Reality," *The Guardian*, 1 October 1998, 7; interview with John Barker.

25 Shehan Hettiaratchy and Peter Butler, “Face Transplantation – Fantasy or Future?” *Lancet* 360; 9326 (2002): 5-6.

26 Hettiaratchy and Butler, “Face Transplantation,” 6. See also Alex Clarke and Peter Butler, “The Psychological Management of Facial Transplantation,” *Expert Review of Neurotherapeutics* 9 (2009): 1087-1100.

27 CNN, “Face Transplant Not Just Science Fiction,” CNN, 28 November 2002, http://edition.cnn.com/2002/HEALTH/11/28/face.transplants/index.html. The work that Butler did at the Royal Free in collaboration with the clinical psychologist Alex Clarke on developing psychological protocols to support face transplant recipients was, and remains, internationally important.

28 https://www.smf.co.uk/publications/science-risk-and-the-media-do-the-front-pages-reflect-reality/.

29 A more detailed study of the UK experience of face transplantation, in relation to the work carried out by Butler and his team at the Royal Free London, is the subject of a separate, in-progress, article.

30 Interview with James Partridge, Director of Face Equality International and founder of Changing Faces, 27 February 2020.

31 Interviews with anonymised participants G06_003 and G01_008

32 Peter J. Morris, J.A. Bradley, L. Doyal, M. Earley, P. Hagan, M. Milling, and N Rumsey, “Facial Transplantation: A Working Party Report from the Royal College of Surgeons of England,” *Transplantation* 77 (2004): 330-338, 330.

33 Ibid., 331-335.

34 Ibid., 337.

35 James Meikle, “Surgeons Warn of Face Transplant Race,” *The Guardian*, 30 November 2003, https://www.theguardian.com/uk/2003/nov/20/education.science.

36 Interview with Professor Richard Ashcroft, formerly ethical advisor to the Royal Free face transplant programme, 23 March 2020.

37 Comité Consultatif National d’Ethique pour les sciences de la vie et de la santé (CNNE), “L’allotransplantation de tissu composite (ATC) au niveau de la face (Greffe totale ou partielle d’un visage),” Paris: Comité consultative national d’éthique pour les sciences de la vie et de la sante, Report No.: Opinion 82 (2004).

38 Xavier Bosch, “Surgeon Denied Ethics Approval for Face Transplantation,” *Lancet* 363; 9412 (2004): 871.

39 Arthur Caplan and Dana Katz, “About Face,” *Hastings Center Report* 33 (2003): 8.

40 Ibid.

41 Jocalyn Clark, “Face Transplants Technically Possible, But ‘Very Hazardous,’” *CMAJ* 170 3 (2004): 323.

42 Meikle, “Surgeons Warn of Face Transplant Race.”

43 Ibid.

44 Saksham Gupta, Ivo S. Muskens, Luis Bradley Fandino, Alexander F.C. Hulsbergen, and Marike L.D. Broekman, "Oversight in Surgical Innovation: A Response to Ethical Challenges," *World Journal of Surgery* 42 (2018): 2773-2780; Allison Hirst, Yiannis Philippou, Jane Blazeby, Bruce Campbell, Marion Campbell, Joshua Feinberg, Maroeska Rovers, Natalie Blencowe, Christopher Pennell, Tom Quinn, Wendy Rogers, Jonathan Cook, Angelos G. Kolias, Riaz Aghu, Philipp Dahm, Art Sedrakyan, Peter McCulloch, "No Surgical Innovation Without Evaluation: Evolution and Further Development of the IDEAL Framework and Recommendations," *Annals of Surgery* 269 (2019): 211-220; Ruth Chadwick and Duncan Wilson, "The Emergence and Development of Bioethics in the UK," *Medical Law Review* 26 (2018): 183–201.

45 Angelique M. Reitsma and Jonathan D. Moreno, “Ethical Regulations for Innovative Surgery: The Last Frontier?” *Journal of the American College of Surgeons* 194 (2002): 792-801.

46 Interview with Ashcroft.

47 M. Joubert, "Chris Barnard: South Africa's Fallible King of Hearts," *South African Journal of Science* 113 (2017): 1-4; Mark Siegler, “Ethical Issues in Innovative Surgery: Should We Attempt a Cadaveric Hand Transplantation in a Human Subject?” *Transplantation Proceedings* 30 (1998): 2279-2782; Rachita Chopra, "The Misconceptions About Surgeons," *Indian Journal of Surgery* 79 (2017): 177.

48 David Concar, “The Boldest Cut,” *New Scientist* 182; 2449 (2004): 32-37.

49 John Barker, Allen Furr, Michael Cunningham, Federico Grossi, Dalibor Vasilic, Barckley Storey, Osborne Wiggins, “Investigation of Risk Acceptance in Facial Transplantation,” *Plastic and Reconstructive Surgery* 118 (2006): 663-670.

50 Rachel A. Ankeny and Ian Kerridge, “On Not Taking Objective Risk Assessments at Face Value,” *American Journal of Bioethics* 4 (2004): 35-37, 36.

51 Raanan Gillon, “Medical Ethics: Four Principles Plus Attention to Scope,” *British Medical Journal* 309; 6948 (1994): 184.

52 Ibid.

53 Lorraine Daston, “Objectivity and the Escape from Perspective,” *Social Studies of Science* 22 (1992): 597-618.

54 Wiggins, Barker, and Martinez, “On the Ethics of Facial Transplantation Research,” 9; Interview with Barker.

55 Concar, “The Boldest Cut.”

56 Delaporte, *Figures of Medicine*, 63.

57 Arthur Caplan, “Facing Ourselves,” *American Journal of Bioethics* 4 (2004): 18-20, 18.

58 *American Journal of Bioethics* 4 (2004): https://www.tandfonline.com/toc/uajb20/4/3.

59 Wiggins, Barker, and Martinez, “On the Ethics of Facial Transplantation Research,” 1.

60 Delaporte, *Figures of Medicine*, 62.

61 Francis Moore, “Three Ethical Revolutions: Ancient Assumptions Remodelled Under Pressure of Transplantation,” *Transplantation Proceedings* 20 (1988): 1061-1067.

62 Siegler, “Ethical Issues in Innovative Surgery.”

63 Wiggins, Barker, and Martinez, “On the Ethics of Facial Transplantation Research,” 8.

64 J.P. Ziegelmann, K. Griva, M. Hankins, M. Harrison, A. Davenport, D. Thompson and S. P. Newman, “The Transplant Effects Questionnaire (TxEQ): The Development of a Questionnaire for Assessing the Multidimensional Outcome of Organ Transplantation,” *British Journal of Health Psychology* 7 (2002): 393-408.

65 Alex Clarke and Peter Butler, “Face Transplantation: Psychological Assessment and Preparation for Surgery,” *Psychology, Health & Medicine* 9 (2004): 315–326.

66 Nichola Rumsey, “Psychological Aspects of Face Transplantation: Read the Small Print Carefully,” *American Journal of Bioethics* 4 (2004): 22-25, 22.

67 Ibid.

68 Wiggins, Barker, and Martinez, “On the Ethics of Facial Transplantation Research,” 5.

69 See also Slatman and Widdershoven, "Hand Transplants and Bodily Integrity."

70 R. Lansdown, N. Rumsey, E. Bradbury, A. Carr, and J. Partridge, ed., *Visibly Different: Coping with Disfigurement* (London: Butterworth-Heinemann, 1997).

71 Samuel Taylor-Alexander, *On Face Transplantation: Life and Ethics in Experimental Biomedicine* (London: Palgrave Macmillan, 2014), 21.

72 Peter Butler, Alex Clarke, and Richard Ashcroft, “Face Transplantation: When and For Whom?” *American Journal of Bioethics* 4 (2004): 16-17.

73 See E. Robinson, N. Rumsey, and J. Partridge, “An Evaluation of the Impact of Social Interaction Skills Training for Facially Disfigured People,” *British Journal of Plastic Surgery* 49 (1996): 281– 289.

74 George J. Agich and Maria Siemionow, “Facing the Ethical Questions in Face Transplantation,” *American Journal of Bioethics* 4 (2004): 25-27, 25.

75 Francois Petit, Antoine Paraskevas, and Laurent Lantieri, “A Surgeons' Perspective on the Ethics of Face Transplantation,” *American Journal of Bioethics* 4 (2004): 14-16, 15.

76 Ibid.

77 Caroline Elton, *Also Human: The Inner Lives of Doctors* (New York: Basic Books, 2018).

78 G.J. Agich and Maria Siemionow, “Until They Have Faces: The Ethics of Facial Allograft Transplantation,” *Journal of Medical Ethics* 31 (2005): 707–709, 707.

79 Ibid., 709.

80 Heather Laine Talley, *Saving Face: Disfigurement and the Politics of Appearance* (New York: New York University Press, 2014), 29-30.

81 Delaporte, *Figures of Medicine*, 65.

82 Thomas Schlich and Christopher Crenner, ed., *Technological Change in Modern Surgery: Historical Perspectives on Innovation* (Rochester: Boydell & Brewer, 2017).

83 Pearl, *Face/On*, 92.

84 Delaporte, *Figures of Medicine*, 65, emphasis in the original.

85 John A. Robertson, “Face Transplants: Enriching the Debate,” *American Journal of Bioethics* 4 (2004): 32-33, 32.

86 This has in fact been the case in several instances. Robertson, “Face Transplants: Enriching the Debate,” 33.

87 Sara Goering, “Facing the Consequences of Facial Transplantation: Individual Choices, Social Effects,” *American Journal of Bioethics* 4 (2004): 37-39, 37.

88 Nuffield Council on Bioethics, *On Cosmetic Procedures: Ethical Issues* (2017).

89 Agich and Siemionow. “Until They Have Faces,” 707.

90 Interview with GO1_005 (anonymised).

91 BBC News, “Woman Has First Face Transplant,” 30 November 2005, http://news.bbc.co.uk/1/hi/health/4484728.stm.

92 Craig S. Smith, “As a Face Transplant Heals, Flurries of Questions Arise,” *New York Times*, 14 December 2005. https://www.nytimes.com/2005/12/14/science/as-a-face-transplant-heals-flurries-of-questions-arise.html.

93 CNNE, Opinion no. 42: "Facial composite tissue allotransplantation (CTA)," 19 February 2002; Personal conversation with Benoît Lengelé, 8 June 2020.

94 Personal conversation with Lengelé; personal conversation with Emmanuel Morelon, 7 July 2020.

95 *Lancet* 368; 9531 (2006): 203–209.

96 P. Petruzzo, S. Testelin, J. Kanitakis, L. Badet, B. Lengelé, J.P. Girbon, and J.M. Dubernard, "First Human Face Transplantation: 5 Years Outcomes," *Transplantation* 93 (2012): 236–240.

97 Ibid.

98 BBC News, “First Face Transplant Patient Isabelle Dinoire Dies in France,” 6 September 2016, https://www.bbc.co.uk/news/world-europe-37290986; Amanda B. Keener, "Saving Face: The Search for Alternatives to Life-Long Immunosuppression for Face Transplants," *Nature Medicine* 22 (2016): 448; personal communication with Morelon.

99 Smith, “As a Face Transplant Heals, Flurries of Questions Arise.”

100 Pearl, *Face/On*, 92.

101 Bound Alberti, “From Face/Off to the Face Race,” 149.

102 Mark Rice-Oxley, “Pet Dog Mauled Face Transplant Patient to Rouse Her From Sleep,” *The Guardian*, 3 December 2005, https://www.theguardian.com/uk/2005/dec/03/health.france.

103 Anthony Barnes and Tom Anderson, “Donor for World’s First Face Transplant Had Hanged Herself,” *The Independent*, 4 December 2005, https://www.independent.co.uk/news/world/europe/donor-for-worlds-first-face-transplant-had-hanged-herself-518079.html.

104 ABC News, “Face Transplant Patient Smoking Again,” 21 August 2006, https://abcnews.go.com/Health/Cosmetic/story?id=1584396&page=1.

105 P. J. Morris, J.A. Bradley, L. Doyal, M. Earley, M. Milling, and N. Rumsey, *Facial Transplantation: Working Party Report*, 2nd Edition (London: Royal College of Surgeons, 2006).

106 “World’s First Full-Face Transplant Likely in UK,” *New Scientist*, 25 October 2006, https://www.newscientist.com/article/dn10374-worlds-first-full-face-transplant-likely-in-uk/.

107 S. Guo, Y. Han, X. Zhang, B. Lu, C. Yi, H. Zhang, and H. Li, “Human Facial Allotransplantation: A 2-year Follow-up Study,” *Lancet* 372*;* 9639 (2008): 631–638.

108 Margaret M. Lock, *Twice Dead: Organ Transplants and Reinvention of Death* (Berkeley: University of California Press, 2002), 71.

109 H.D. Kvernmo, V.S. Gorantla, and R.N. Gonzalez, “Hand Transplantation,” *Acta Orthopaedica* 76 (2005): 14–27.

110 S.D. Mendenhall, S. Brown, O. Ben-Amotz, M.W. Neumeister, and L.S. Levin, “Building a Hand and Upper Extremity Transplantation Program: Lessons Learned From the First 20 Years of Vascularized Composite Allotransplantation,” *HAND* 15 (2020): 224–233.

111 T. Garrel-Jaffrelot, “Frenchman Is First in World to Get 2 Full Face Transplants,” *New York Times*, 19 April 2018, https://www.nytimes.com/2018/04/19/world/europe/jerome-hamon-face-transplant-france.html.

112 Interview with Barker; Interview with Clarke.

113 Ed Pilkington, “US Face Transplant Recipient Makes Public Appearance,” *The Guardian*, 6 May 2009, https://www.theguardian.com/world/2009/may/06/connie-culp-face-transplant-usa.

114 Maria Siemionow, Frank Papay, D. Alam, S. Bernard, R. Djohan, C. Gordon, and J. Fung, “Near-Total Human Face Transplantation for a Severely Disfigured Patient in the USA,” *Lancet* 374*;* 9685 (2009): 203–209.

115 “Face Transplant Recipient Connie Culp Meets Donor Family,” *ABC News*, 21 December 2010, https://www.youtube.com/watch?v=pbR1lMIcLuQ.

116 Fay Bound Alberti and Victoria Hoyle, "First Face Transplant Recipient Dies, Leaving an Important Legacy," *The Conversation*, 18 August 2020, https://theconversation.com/first-us-face-transplant-recipient-dies-leaving-an-important-legacy-144522.

117 Joanna Connors, “How a Face Transplant Transformed a Young Woman’s Life,” *National Geographic*, September 2018, https://www.nationalgeographic.com/magazine/2018/09/face-transplant-katie-stubblefield-story-identity-surgery-science/; BBC World Service, “Outlook, The Story of My Face Transplant,” 22 January 2019, https://www.bbc.co.uk/programmes/w3cswkzg.

118 M.W. McQuinn, L.L. Kimberly, B. Parent, J. Rodrigo Diaz-Siso, A.L. Caplan, A.G. Blitz, and E.D. Rodriguez, “Self-Inflicted Gunshot Wound as a Consideration in the Patient Selection Process for Facial Transplantation,” *Cambridge Quarterly of Healthcare Ethics* 28 (2019): 450–462.

119 G. Giatsidis, I. Sinha, and B. Pomahac, “Reflections on a Decade of Face Transplantation,” *Annals of Surgery* 265 (2017): 841-846.

120 Brigham and Women’s Hospital, “DoD Funding for Face Transplant Study,” n.d., https://www.brighamandwomens.org/surgery/plastic-surgery/restorative-surgery/dod-study; The Cleveland Clinic, “Face Transplants: The Changing Face of Medicine,” 2018, https://newsroom.clevelandclinic.org/2018/08/14/face-transplants-the-changing-face-of-medicine/.

121 Interview with Barker.

122 Laurent Lantieri, P. Grimbert, N. Ortonne, C. Suberbielle, D. Bories, S. Gil-Vernet, and M. Hivelin, “Face Transplant: Long-term Follow-up and Results of a Prospective Open Study,” *Lancet* 388; 10052 (2016): 1398–1407.

123 J. Hamon, T’as vu le monsieur? (Flammarion, 2015).

124 P. Petruzzo, J. Kanitakis, S. Testelin, J.B. Pialat, F. Buron, L. Badet, and E. Morelon, “Clinicopathological Findings of Chronic Rejection in a Face Grafted Patient,” *Transplantation* 99 (2015): 2644–2650.

125 Ö. Özkan, Ö. Özkan, M. Ubur, N. Hadimioğlu, M. Cengiz, and I. Afşar, “Face Allotransplantation for Various Types of Facial Disfigurements: A Series of Five Cases,” *Microsurgery* 38 (2018): 834–843.

126 “Turkish Transplant Patient Sees Face for First Time,” *The Telegraph*, 13 February 2012, https://www.telegraph.co.uk/news/worldnews/europe/turkey/9079201/Turkish-transplant-patient-sees-face-for-first-time.html; “Turkey’s Fourth Face Transplant Patient Ties the Knot,” *Daily Sabah*, September 2015, https://www.dailysabah.com/life/2015/09/10/turkeys-fourth-face-transplant-patient-ties-the-knot.

127 L. Lantieri, et al. “Face Transplant: Long-Term Follow-Up.”

128 W.S. Cherikh, L.C. Cendales, C. L. Wholley, J. Wainright, V.S. Gorantla, D.K. Klassen, and L. Scott Levin, “Vascularized Composite Allotransplantation in the United States: A Descriptive Analysis of the Organ Procurement and Transplantation Network Data,” *American Journal of Transplantation* 19 (2019): 865–875.

129 We are not able to confirm these assumptions, as the waiting list is confidential.

130 Interview with Clarke.

131 Helen Puttick, “Surgeon Frustrated at Face Transplant Snub by NHS,” *The Herald*, 29 January 2016, https://www.heraldscotland.com/news/14238822.surgeon-frustrated-at-face-transplant-snub-by-nhs/.

132 Arthur Caplan and D. Purve, “A Quiet Revolution in Organ Transplant Ethics,” *Journal of Medical Ethics 43* (2017): 797-800.

133 E. Rosenberg, “Face Transplant: Canadian Hunter Maurice Desjardins Becomes World’s Oldest Face Transplant Recipient,” *Washington Post*, 14 September 2018, https://www.washingtonpost.com/news/to-your-health/wp/2018/09/14/his-face-was-severely-damaged-on-a-hunt-now-hes-the-worlds-oldest-face-transplant-recipient/; J. Ducharme, “NYU Performs Its 2nd Face Transplant on Cameron Underwood,” *Time*, November 2018, https://time.com/5466810/cameron-underwood-face-transplant/.

134 Private correspondence with Andrew Lingford and Benita Antas, Helsinki University Hospital, 25 February 2020. It is worth noting that the media attention and interest seen elsewhere, in the UK and the US, does not apply, which lessens the negative impact on families and patients.

135 Ibid.

136 L. Mazzone, M. Pratsinis, L. Pontiggia, E. Reichmann, and M. Meuli, “Successful Grafting of Tissue-Engineered Fetal Skin,” *Pediatric Surgery International* 32 (2016): 1177-1182.

137 Delaporte, *Figures of Medicine*, 66.

138 Interview with G06_004 (anonymous interviewee), 19 May 2020.

139 Anne F. Klassen, Stefan J. Cano, Amie Scott, Laura Snell, and Andrea L. Pusic, "Measuring Patient-Reported Outcomes in Facial Aesthetic Patients: Development of the FACE-Q," *Facial Plastic Surgery* 26 (2010): 303.

140 For example, Ericka M. Bueno, J. Rodrigo Diaz-Siso, and Bohdan Pomahac, “A Multidisciplinary Protocol for Face Transplantation at Brigham and Women’s Hospital,” *Journal of Plastic, Reconstructive & Aesthetic Surgery* 64 (2011): 1572-1579; L.L. Kimberly, A.R. Alfonso, E.P. Ramly, R.S. Kantar, A.L. Caplan, and E. D. Rodriguez, “How to Integrate Lived Experience Into Quality-of-Life Assessment in Patients Considering Facial Transplantation,” *AMA Journal of Ethics* 21 (2019): 980-987; Emily Herrington, "What’s Missing in Our Thinking About Quality of Life in VCA?" *AMA Journal of Ethics* 21 (2019): 968-973; Emily Herrington and Jessica Benham, "Assessing Quality of Life in Reconstructive Transplantation," *AMA Journal of Ethics* 21 (2019): 933-935; J. Benedict and G. Magill, "Ethics and the Future of Vascularized Composite Allotransplantation," *Current* *Transplantation Reports* 5 (2018): 334-338.

141 A.L. Caplan et al, “Emerging Ethical Challenges Raised by the Evolution of Vascularized Composite Allotransplantation.”

142 Interview with Clarke; interview with Barker.

