# Supplementary material for: Face Transplants: An International History
Source: J Hist Med Allied Sci. 2021 Jun 28;76(3):319–45. doi: 10.1093/jhmas/jrab019 (PMC8420670; doi:10.1093/jhmas/jrab019)
Supplement: jrab019_Supplementary_Data [file jrab019_supplementary_data.docx]

| Name | Type of transplant | Venue | Country | Date | Gender | Age at time of transplant | Age at time of facial change | Cause of facial difference |
| --- | --- | --- | --- | --- | --- | --- | --- | --- |
| Dinoire, Isabelle^[[1]](#endnote-1)^ ^[[2]](#endnote-2)^  (d. April 2016)^[[3]](#endnote-3)^ | Partial | Centre hospitalier Universitaire Nord, Amiens | France | 27 Nov 2005 | Female | 38 | 38 | Mauled by pet dog following sleeping tablet overdose^[[4]](#endnote-4)^ |
| Guoxing, Li^[[5]](#endnote-5)^  (d. July 2008)^[[6]](#endnote-6)^ | Partial | Xijing Military Hospital, Xi'an | China | 13 Apr 2006 | Male | 30 | 28 | Attacked by bear |
| Coler, Pascale^[[7]](#endnote-7)^ | Partial | Henri Mondor Hospital, Paris | France | 21 Jan 2007 | Male | 29 | Genetic | Neurofibromatosis type 1 |
| Culp, Connie^[[8]](#endnote-8)^ ^[[9]](#endnote-9)^  (d. 29 July 2020)^[[10]](#endnote-10)^ | Partial | Cleveland Clinic, Ohio | USA | 9 Dec 2008 | Female | 45 | 41 | Third party gunshot injury |
| (Anonymous)^[[11]](#endnote-11)^ | Partial | Henri Mondor Hospital, Paris | France | 24 Mar 2009 | Male | 27 | 23^[[12]](#endnote-12)^ | Accidental gunshot injury^[[13]](#endnote-13)^ |
| (Anonymous) ^[[14]](#endnote-14)^  (d. 8 June 2009) | Partial  (& bi-lateral hand) | Henri Mondor Hospital, Paris | France | 4 Apr 2009 | Male | 37 | 33 | Self-immolation^[[15]](#endnote-15)^ |
| Maki, James^[[16]](#endnote-16)^ | Partial | Brigham and Woman's Hospital, Boston | USA | Apr 2009^[[17]](#endnote-17)^ | Male | 59 | 55^[[18]](#endnote-18)^ | Electrical burns from accident |
| (Anonymous)^[[19]](#endnote-19)^  (d. by June 2013)^[[20]](#endnote-20)^ | Partial | Hospital La Fe, Valencia | Spain | Aug 2009 | Male | 42 | 31 | Tongue cancer |
| (Anonymous) ^[[21]](#endnote-21)^ | Partial | Henri Mondor Hospital, Paris | France | 19 Aug 2009 | Male | 33 | 29/30^[[22]](#endnote-22)^ | Self-inflicted gunshot injury |
| (Anonymous)^[[23]](#endnote-23)^  (d. after 2014)^[[24]](#endnote-24)^ | Partial | Centre hospitalier Universitaire Nord, Amiens | France | 27 Nov 2009 | Male | 27 | 26 | Pyrotechnic explosion |
| Rafael^[[25]](#endnote-25)^ | Partial | Virgen del Rocio Hospital, Seville | Spain | 26 Jan 2010 | Male | 35 | Genetic | Neurofibromatosis type 1 |
| Oscar^[[26]](#endnote-26)^ | Full | Vall d'Hebron Hospital, Barcelona | Spain | 27 Mar 2010 | Male | 31^[[27]](#endnote-27)^ | 26 | Gunshot injury |
| Hamon, Jérôme^[[28]](#endnote-28)^ | Full | Henri Mondor Hospital, Paris | France | 27 June 2010 | Male | 35 | Genetic | Neurofibromatosis type 1 |
| Wiens, Dallas^[[29]](#endnote-29)^ | Full | Brigham and Woman's Hospital, Boston | US | March 2011^[[30]](#endnote-30)^ | Male | 25 | 22 | Electrical burns from accident |
| (Anonymous)^[[31]](#endnote-31)^ | Partial | Henri Mondor Hospital, Paris | France | 2011 | Male | 45 | 43 | Accidental gunshot injury |
| (Anonymous)^[[32]](#endnote-32)^  (d. 2014) | Partial | Henri Mondor Hospital, Paris | France | April 2011 | Male | 41 | 35 | Self-inflicted gunshot injury |
| Hunter, Mitch^[[33]](#endnote-33)^ | Full | Brigham and Woman's Hospital, Boston | US | April 2011 | Male | 30 | 21^[[34]](#endnote-34)^ | Electrical burns from road traffic accident |
| Nash, Charla^[[35]](#endnote-35)^ | Full  (& failed bilateral hand) | Brigham and Woman's Hospital, Boston | US | May 2011 | Female | 57^[[36]](#endnote-36)^ | 54 | Mauled by a chimpanzee |
| (Anonymous)^[[37]](#endnote-37)^ | Partial | University Hospital, Ghent | Belgium | 2011 | Male | 54 | ? | Gunshot injury |
| Acar, Ugur^[[38]](#endnote-38)^ ^[[39]](#endnote-39)^ | Full | Akdeniz University School of Medicine | Turkey | 21 Jan 2012 | Male | 19 | 1 month | Burns from a domestic fire |
| Gül, Cengiz^[[40]](#endnote-40)^ | Full | Hacettepe University | Turkey | 24 Feb 2012 | Male | 25 | 2 | Electrical burns from accident |
| Nergis, Hatice^[[41]](#endnote-41)^  (d. Nov 2016)^[[42]](#endnote-42)^ | Partial | Gazi University Hospital, Ankara | Turkey | 17 Mar 2012 | Female | 20 | 14 | Gunshot injury |
| Norris, Richard^[[43]](#endnote-43)^ ^[[44]](#endnote-44)^ | Full | University of Maryland Medical Center, Baltimore | US | 19 Mar 2012 | Male | 37 | 22 | Accidental gunshot injury |
| Çolak, Turan^[[45]](#endnote-45)^ ^[[46]](#endnote-46)^ | Full | Akdeniz University School of Medicine | Turkey | 15 May 2012 | Male | 35 | 3 | Burns from domestic accident |
| (Anonymous)^[[47]](#endnote-47)^ | Partial | Centre hospitalier Universitaire Nord, Amiens | France | June 2012 | Female | 52 | Progressive | Vascular tumour |
| Tarleton, Carmen Blandin^[[48]](#endnote-48)^ ^[[49]](#endnote-49)^ | Full | Brigham and Woman's Hospital, Boston | US | 14 Feb 2013 | Female | 44 | 39 | Chemical burns from domestic abuse attack |
| Galasiński, Grzegorz^[[50]](#endnote-50)^ ^[[51]](#endnote-51)^ | Partial | Maria Skłodowska-Curie Institute of Oncology, Gliwice | Poland | 15 May 2013 | Male | 31/33^[[52]](#footnote-1)^ | 31 | Industrial accident |
| Sert, Recep^[[53]](#endnote-52)^ ^[[54]](#endnote-53)^ | Full | Akdeniz University School of Medicine | Turkey | 18 July 2013 | Male | 26 | 20 | Gunshot injury |
| Üstün, Salih^[[55]](#endnote-54)^ ^[[56]](#endnote-55)^  (d. July 2014)^[[57]](#endnote-56)^ | Full | Akdeniz University School of Medicine | Turkey | 23 Aug 2013 | Male | 54 | 50 | Gunshot injury |
| Joanna^[[58]](#endnote-57)^ ^[[59]](#endnote-58)^ | Partial | Maria Skłodowska-Curie Institute of Oncology, Gliwice | Poland | 4 Dec 2013 | Female | 26/29^[[60]](#footnote-2)^ | Genetic | Neurofibromatosis type 1 |
| Kaya, Recep^[[61]](#endnote-59)^ ^[[62]](#endnote-60)^ | Partial | Akdeniz University School of Medicine | Turkey | 28 Dec 2013 | Male | 22 | 17 | Gunshot injury |
| (Anonymous)^[[63]](#endnote-61)^ | Partial | Brigham and Woman's Hospital, Boston | US | March 2014 | Male | 39 | ? | Gunshot injury |
| Fiddler, Shaun^[[64]](#endnote-62)^ ^[[65]](#endnote-63)^ | Partial | Cleveland Clinic, Ohio | US | Sept 2014 | Male | 46 | 43 | Road traffic accident |
| (Anonymous)^[[66]](#endnote-64)^ | Partial | Brigham and Woman's Hospital, Boston | US | October 2014 | Male | 33 | ? | Gunshot injury |
| (Anonymous)^[[67]](#endnote-65)^ | Full | Vall d'Hebron Hospital, Barcelona | Spain | Jan-Mar 2015 | Male | 45 | Progressive | Arteriovenous Malformation |
| (Anonymous)^[[68]](#endnote-66)^ | Partial | S.M.Kirov Military Medical Academy, St Petersburg | Russia | May 2015 | Male | 21/22 | 19 | Electrical burns |
| Hardison, Patrick^[[69]](#endnote-67)^ | Full | NYU Langone Medical Center, New York | US | 14-15 Aug 2015 | Male | 41 | 27 | Burns received as a firefighter |
| (Anonymous)^[[70]](#endnote-68)^ | Partial | Helsinki University Hospital | Finland | 8 Feb 2016 | Male | 34 | 17 | Gunshot injury |
| Sandness, Andrew^[[71]](#endnote-69)^ ^[[72]](#endnote-70)^ | Full | Mayo Clinic, Rochester, Minnesota | US | June 2016 | Male | 31 | 21 | Gunshot injury |
| Stubblefield, Katie^[[73]](#endnote-71)^ | Full | Cleveland Clinic, Ohio | US | 4 May 2017 | Female | 21 | 17 | Self-inflicted gunshot injury |
| Underwood, Cameron^[[74]](#endnote-72)^ ^[[75]](#endnote-73)^ | Partial | NYU Langone Medical Center, New York | US | 5-6 Jan 2018 | Male | 26 | 24 | Self-inflicted gunshot injury |
| (Anonymous)^[[76]](#endnote-74)^ | Full | Helsinki University Hospital | Finland | March 2018 | Male | 58 | ? | Gunshot injury |
| Hamon, Jérôme^[[77]](#endnote-75)^ | Full (retransplant) | Georges-Pompidou European Hospital, Paris | France | April 2018 | Male | 43 | Genetic | First transplant failed after rejection |
| Desjardins, Maurice^[[78]](#endnote-76)^ | Partial | Hopital Maisonneuve Rosemont, Montreal, Quebec | Canada | May 2018 | Male | 64 | 57 | Gunshot injury |
| (Anonymous)^[[79]](#endnote-77)^ | Partial  (failed) | Sant' Andrea Hospital, Sapienza University, Rome | Italy | 22-23 Sept 2018 | Female | 49 | Genetic | Neurofibromatosis type 1 |
| Chelsea, Robert^[[80]](#endnote-78)^ | Full | Brigham and Woman's Hospital, Boston | US | July 2019 | Male | 68 | 62 | Burns received in a car fire |
| Tarleton, Carmen Blandin^[[81]](#endnote-79)^ | Full  (retransplant) | Brigham and Women’s Hospital, Boston | USA | July 2020 | Female | 52 | 39 | First transplant failed after chronic rejection |

1. Devauchelle et al., “First Human Face Allograft: Early Report.” [↑](#endnote-ref-1)
2. Petruzzo et al., “First Human Face Transplantation: 5 Years Outcomes.” [↑](#endnote-ref-2)
3. BBC News, “First Face Transplant Patient Isabelle Dinoire Dies in France.” [↑](#endnote-ref-3)
4. Rice-Oxley, “Pet dog mauled face-transplant patient to rouse her from sleep.” [↑](#endnote-ref-4)
5. Guo et al., “Human Facial Allotransplantation.” [↑](#endnote-ref-5)
6. Jordan Lite, “Chinese Face Transplant Recipient Has Died,” *Scientific American* (22 December 2008), <https://blogs.scientificamerican.com/news-blog/chinese-face-transplant-recipient-h-2008-12-22/>. [↑](#endnote-ref-6)
7. L. Lantieri, J.P. Meningaud, P. Grimbert, F. Bellivier, J.P. Lefaucheur, N. Ortonne, and P. Wolkenstein, “Repair of the Lower and Middle Parts of the Face by Composite Tissue Allotransplantation in a Patient with Massive Plexiform Neurofibroma: A 1-year Follow-up Study. *Lancet* 372; 9639 (2008): 639–645. [↑](#endnote-ref-7)
8. Siemionow et al., “Near-Total Human Face Transplantation,” 203–209. [↑](#endnote-ref-8)
9. W. Bergfeld, A. Klimczak, J.S. Stratton, and M.Z. Siemionow, “A Four-Year Pathology Review of the Near Total Face Transplant,” *American Journal of Transplantation* 13 (2013): 2750-2764. [↑](#endnote-ref-9)
10. Artemis Moshtaghian and Dakin Andone, “Connie Culp, the First Person to Receive a Near-Total Face Transplant in the USA, Dies,” *CNN* (1 August 2020), <https://edition.cnn.com/2020/08/01/us/face-transplant-connie-culp-dies-trnd/index.html>. [↑](#endnote-ref-10)
11. L. Lantieri, M. Hivelin, V. Audard, M.D. Benjoar, J.P. Meningaud, F. Bellivier, and P. Grimbert, “Feasibility, Reproducibility, Risks and Benefits of Face Transplantation: A Prospective Study of Outcomes,” *American Journal of Transplantation* 11 (2011): 367–378. [↑](#endnote-ref-11)
12. Lantieri et al., “Face Transplant: Long-term Follow-up.” [↑](#endnote-ref-12)
13. Ibid. [↑](#endnote-ref-13)
14. Lantieri et al., “Feasibility, Reproducibility, Risks and Benefits of Face Transplantation.” [↑](#endnote-ref-14)
15. Lantieri et al., “Face Transplant: Long-term Follow-up.” [↑](#endnote-ref-15)
16. B. Pomahac, J. Pribaz, E. Eriksson, D. Annino, S. Caterson, C. Sampson, and S.G. Tullius, “Restoration of Facial Form and Function After Severe Disfigurement from Burn Injury by a Composite Facial Allograft,” *American Journal of Transplantation* 11 (2011): 386–393. [↑](#endnote-ref-16)
17. J.R. Diaz-Siso, M. Parker, E.M. Bueno, G.C. Sisk, J.J. Pribaz, E. Eriksson, and B. Pomahac, “Facial Allotransplantation: A 3-year Follow-Up Report,” *Journal of Plastic, Reconstructive and Aesthetic Surgery* 66 (2013): 1458–1463. [↑](#endnote-ref-17)
18. “Patient’s Story: Jim Maki’s Face Transplant,” (n.d.), *Brigham and Women’s Hospital*, <https://www.brighamandwomens.org/life-giving-breakthroughs/patient-stories/jim>. [↑](#endnote-ref-18)
19. P.C. Cavadas, J. Ibez, and A. Thione, “Surgical Aspects of a Lower Face, Mandible, and Tongue Allotransplantation,” *Journal of Reconstructive Microsurgery* 28 (2012): 43–47. [↑](#endnote-ref-19)
20. Anna Cuenca, “First Double-Leg Transplant Patient Has Limbs Amputated (Update),” *Medical Xpress.com*, 1 June 2013, <https://medicalxpress.com/news/2013-06-double-leg-transplant-patient-limbs-amputated.html>. [↑](#endnote-ref-20)
21. Lantieri et al., “Feasibility, Reproducibility, Risks and Benefits of Face Transplantation.” [↑](#endnote-ref-21)
22. Lantieri et al., “Face transplant: Long-Term Follow-Up.” [↑](#endnote-ref-22)
23. Petruzzo et al., “Clinicopathological Findings of Chronic Rejection in a Face Grafted Patient.” [↑](#endnote-ref-23)
24. Interview with Professor Emmanuel Morelon, 8 July 2020. [↑](#endnote-ref-24)
25. D. Sicilia-Castro, T. Gomez-Cia, P. Infante-Cossio, P. Gacto-Sanchez, F. Barrera-Pulido, A. Lagares-Borrego, and J.D. Gonzalez-Padilla, “Reconstruction of a Severe Facial Defect by Allotransplantation in Neurofibromatosis Type 1: A Case Report,” *Transplantation Proceedings* 43 (2011): 2831–2837. [↑](#endnote-ref-25)
26. J.P. Barret, J. Serracanta, J. M. Collado, A. Garrido, P. Salamero, T. Pont, and M. Ruiz, “Full Face Transplantation Organization, Development, and Results-The Barcelona Experience: A Case Report,” *Transplantation Proceedings* 43 (2011): 3533–3534. [↑](#endnote-ref-26)
27. BBC News, “Full Face Transplant Patient Reveals His New Look on TV,” *BBC News*, 26 July 2010, <https://www.bbc.co.uk/news/health-10765005>. [↑](#endnote-ref-27)
28. Lantieri et al., “Face Transplant: Long-Term Follow-Up.” [↑](#endnote-ref-28)
29. B. Pomahac, J. Pribaz, E. Eriksson, E.M. Bueno, J.R. Diaz-Siso, F.J. Rybicki, and S.G. Tullius, “Three Patients with Full Facial Transplantation,” *New England Journal of Medicine* 366 (2012): 715–722. [↑](#endnote-ref-29)
30. Katie Moisse, “Dallas Wiens Smiling 10 Months After Full Face Transplant,” ABCNews, 15 January 2012, <https://abcnews.go.com/Health/Wellness/dallas-wiens-smiling-full-face-transplant/story?id=15366582>. [↑](#endnote-ref-30)
31. Lantieri et al., “Face Transplant: Long-Term Follow-Up.” [↑](#endnote-ref-31)
32. Ibid. [↑](#endnote-ref-32)
33. Pomahac et al., “Three Patients with Full Facial Transplantation.” [↑](#endnote-ref-33)
34. Mia de Graaf, “Ex-Soldier Who Lost His Face Saving Woman's Life in Horrific Accident Reveals Remarkable Transformation After Receiving One of the World's First Ever Transplants,” *The Daily Mail*, 19 April 2016,

    <https://www.dailymail.co.uk/health/article-3546860/It-stronger-Former-soldier-received-one-world-s-face-transplant-reveals-remarkable-transformation-insists-s-never-felt-better.html>. [↑](#endnote-ref-34)
35. Pomahac et al., “Three Patients with Full Facial Transplantation.” [↑](#endnote-ref-35)
36. The Associated Press, “Mauled Woman Gets Full Face Transplant,” *New York Times*, 11 June 2011, <https://www.nytimes.com/2011/06/11/nyregion/charla-nash-attacked-by-chimp-gets-full-face-transplant.html>. [↑](#endnote-ref-36)
37. N.A. Roche, H.F. Vermeersch, F.B. Stillaert, K.T. Peters, J. De Cubber, K. Van Lierde, and P.N. Blondeel, “Complex Facial Reconstruction by Vascularized Composite Allotransplantation: The First Belgian Case,” *Journal of Plastic, Reconstructive and Aesthetic Surgery* 68 (2015): 362–371. [↑](#endnote-ref-37)
38. Ö. Özkan, Ö. Özkan, M. Ubur, N. Hadimioğlu, M. Cengiz, and İ. Afşar, “Face Allotransplantation for Various Types of Facial Disfigurements: A Series of Five Cases,” *Microsurgery* 38 (2018): 834–843. [↑](#endnote-ref-38)
39. “Turkish Transplant Patient Sees Face For First Time,” *The Telegraph*, 13 February 2012. <https://www.telegraph.co.uk/news/worldnews/europe/turkey/9079201/Turkish-transplant-patient-sees-face-for-first-time.html>. [↑](#endnote-ref-39)
40. “Turkish Surgeons Complete a Series of Miraculous Full-Face Transplants,” *International Medical Tourism Webportal*, n.d. <http://www.intmedtourism.com/en/news/443.html>. [↑](#endnote-ref-40)
41. Ibid. [↑](#endnote-ref-41)
42. "Türkiye'nin yüz nakli yapılan ilk kadını Hatice Nergis öldü," *Hürriyet* (in Turkish; translated with Google Translate), 15 November 2016. <http://www.hurriyet.com.tr/turkiyenin-yuz-nakli-yapilan-ilk-kadini-hatice-nergis-oldu-40278523>. [↑](#endnote-ref-42)
43. Amir H. Dorafshar, Branko Bojovic, Michael R. Christy, Daniel E. Borsuk, Nicholas T. Iliff, Emile N. Brown, Cynthia K. Shaffer, T. Nicole Kelley, Debra L. Kukuruga, Rolf N. Barth, Stephen T. Bartlett and Eduardo D. Rodriguez, “Total Face, Double Jaw, and Tongue Transplantation: An Evolutionary Concept,” *Plastic and Reconstructive Surgery* 131 (2013): 241-251. [↑](#endnote-ref-43)
44. Jeanne Marie Laskas, “The New Face of Richard Norris,” *GQ*, 28 August 2014, <https://www.gq.com/story/richard-norris>. [↑](#endnote-ref-44)
45. Özkan et al, “Face Allotransplantation for Various Types of Facial Disfigurements.” [↑](#endnote-ref-45)
46. “Turkish Surgeons Complete a Series of Miraculous Full-Face Transplants.” [↑](#endnote-ref-46)
47. B.L. Devauchelle, S.R. Testelin, J. Davrou, E. Neiva, B.G. Lengele, J-M. Dubernard, and S. Ephanie Dakpe, “Face Graft? Extrapolation of Facial Allotransplantation to Children,” *Journal of Cranio-Maxillo-Facial Surgery* 44 (2016): 925–933. [↑](#endnote-ref-47)
48. Branislav Kollar and Bohdan Pomahac, “Facial Restoration by Transplantation,” *Surgeon* 16 (2018): 245-249. [↑](#endnote-ref-48)
49. K. Drummond, “Beyond Recognition: The Incredible Story of a Face Transplant,” *The Verge*, 4 June 2013, <https://www.theverge.com/2013/6/4/4381890/carmen-tarleton-the-incredible-story-of-a-face-transplant>. [↑](#endnote-ref-49)
50. A. Maciejewski, L. Krakowczyk, C. Szymczyk, J. Wierzgon, M. Grajek, M. Dobrut, and S. Poltorak, “The First Immediate Face Transplant in the World,” *Annals of Surgery* 262 (2016): e36-e39. [↑](#endnote-ref-50)
51. Tara Brady, “The Incredible Results of Polish Surgeons' First Face Transplant on Man who was Injured by Stone-Cutting Machine,” *The Daily Mail*, 30 May 2013, <https://www.dailymail.co.uk/news/article-2333490/The-incredible-results-Polish-surgeons-face-transplant-man-injured-stone-cutting-machine.html>. [↑](#endnote-ref-51)
52. Galasiński’s age is consistently reported as 33 in the popular press and in previous face transplant surveys, but as 31 in the published case report. [↑](#footnote-ref-1)
53. Özkan et al., “Face Allotransplantation for Various Types of Facial Disfigurements.” [↑](#endnote-ref-52)
54. “Turkey’s Fifth Face Transplant Patient Sees Face for First Time Since Surgery,” *Hurriyet*, 5 September 2013, <https://www.hurriyetdailynews.com/turkeys-fifth-face-transplant-patient-sees-face-for-first-time-since-surgery-53915>. [↑](#endnote-ref-53)
55. Özkan et al., “Face Allotransplantation for Various Types of Facial Disfigurements.” [↑](#endnote-ref-54)
56. “Turkey’s 6th Transplant Performed in Antalya,” *Hurriyet*, 23 August 2013, <https://www.hurriyetdailynews.com/turkeys-6th-transplant-performed-in-antalya-53102>. [↑](#endnote-ref-55)
57. Özkan et al., “Face Allotransplantation for Various Types of Facial Disfigurements.” [↑](#endnote-ref-56)
58. I. Makles-Kacy, E. Zys-Owczarek, A. Polak, and A. Maciejewski, “Physiotherapy in the First Three Months After Allograft Face Transplantation – Case Report,” *Physiotherapy* 24 (2016): 34–38. [↑](#endnote-ref-57)
59. “Second Face Transplant in Poland ‘A Success,’” *Radio Poland*, 12 December 2013, <http://archiwum.thenews.pl/1/9/Artykul/155818,Second-face-transplant-in-Poland-a-success>. [↑](#endnote-ref-58)
60. Joanna’s age is frequently reported as 26 in the media reporting immediately after her transplant, but as 29 in the published literature. [↑](#footnote-ref-2)
61. Özkan et al., “Face Allotransplantation for Various Types of Facial Disfigurements.” [↑](#endnote-ref-59)
62. “Face Transplant Patient Enjoys His ‘New Life,’” *Daily Sabah*, 16 April 2014, <https://www.dailysabah.com/health/2014/04/16/face-transplant-patient-enjoys-their-new-life>. [↑](#endnote-ref-60)
63. Kollar and Pomahac, “Facial Restoration by Transplantation.” [↑](#endnote-ref-61)
64. Marilynn Marchione, “Cleveland Clinic Does its Second Face Transplant,” *Medical Xpress*, 18 November 2014, <https://medicalxpress.com/news/2014-11-cleveland-clinic-transplant.html>. [↑](#endnote-ref-62)
65. Martin Schoeller and Joanna Connors, “What’s It Like to Have a New Face? Transplant Recipients Tell Us,” *National Geographic*, September 2018, <https://www.nationalgeographic.com/magazine/2018/09/face-transplant-recipients-profiles/>. [↑](#endnote-ref-63)
66. Kollar and Pomahac, “Facial Restoration by Transplantation.” [↑](#endnote-ref-64)
67. Alistair Dawber, “Spain’s Pioneering Vall d’Hebron Hospital Rebuilds Man’s Face and Neck During Complex 27 Hour Operation,” *The Independent*, 31 March 2015, <https://www.independent.co.uk/life-style/health-and-families/health-news/spains-pioneering-vall-dhebron-hospital-rebuilds-mans-face-and-neck-during-complex-27-hour-operation-10147420.html>. [↑](#endnote-ref-65)
68. Maria Volokh, N. Manturova, A. Fisun, V. Uyba, S. Voskanyan, G. Khubulava, N. Kalakutskiy, and K. Gubarev, “First Russian Experience of Composite Facial Tissue Allotransplantation,” *Plastic and Reconstructive Surgery - Global Open* 7 (2019): e2521. [↑](#endnote-ref-66)
69. J.R. Diaz-Siso, M. Sosin, N.M. Plana, and E.D. Rodriguez, “Face Transplantation: Complications, Implications, and an Update for the Oncologic Surgeon,” *Journal of Surgical Oncology* 113 (2016): 971–975. [↑](#endnote-ref-67)
70. Patrik Lassus, Andrew Lindford, Jyrki Vuola, Leif Bäck, Sinikka Suominen, Karri Mesimäki, Tommy Wilkman, Tuija Ylä-Kotola, Erkki Tukiainen, Hannu Kuokkanen, and Jyrki Törnwall, “The Helsinki Face Transplantation: Surgical Aspects and 1-year Outcome,” *Journal of Plastic, Reconstructive and Aesthetic Surgery* 71 (2018): 132–139. [↑](#endnote-ref-68)
71. H. Amer, S. Jowsey-Gregoire, C.B. Rosen, M. Gandhi, B.S. Edwards, L.E. Ewoldt, and S. Mardini, “Mayo Clinicʼs First Face Transplant,” *Transplantation* 102 (2018): S433. [↑](#endnote-ref-69)
72. Hannah Tiede, “Face Transplant Recipient Transforming Lives with a Message of Hope,” *ABC6News*, 26 September 2019, <https://www.kaaltv.com/news/transform-conference-mayo-clinic-face-transplant-andy-sandness-first-latest-rochester-mn/5507508/>. [↑](#endnote-ref-70)
73. Joanna Connors, “How a Face Transplant Transformed a Young Woman’s Life,” *National Geographic*, September 2018, <https://www.nationalgeographic.com/magazine/2018/09/face-transplant-katie-stubblefield-story-identity-surgery-science/>. [↑](#endnote-ref-71)
74. Rami S. Kantar, Daniel J. Ceradini, Bruce E. Gelb, Jamie P. Levine, David A. Staffenberg, Pierre B. Saadeh, Roberto L. Flores, Nicole G. Sweeney, G. Leslie Bernstein, and Eduardo D. Rodriguez, “Facial Transplantation for an Irreparable Central and Lower Face Injury: A Modernized Approach to a Classic Challenge,” *Plastic and Reconstructive Surgery* 144 (2019): 264e-283e. [↑](#endnote-ref-72)
75. Jeff Nelson, “California Man, 26, Receives Face Transplant After Failed Suicide Attempt,” *People*, 14 November 2018, <https://people.com/human-interest/cameron-underwood-nyu-langone-face-transplant-after-suicide-attempt/>. [↑](#endnote-ref-73)
76. Andrew J. Lindford, Heikki Mäkisalo, Hannu Jalanko, Jouni Lauronen, Veli-Jukka Anttila, Susanna Juteau, Antti-Jussi Ämmälä, Anna Eskola, Samuli Saarni, Helena Isoniemi, Antti Mäkitie, and Patrik Lassus, “The Helsinki Approach to Face Transplantation,” *Journal of Plastic, Reconstructive and Aesthetic Surgery* 72 (2019): 173–180. [↑](#endnote-ref-74)
77. Garrel-Jaffrelot, “Frenchman Is First in World to Get 2 Full Face Transplants.” [↑](#endnote-ref-75)
78. S. Kirkey, “Canada’s First Face Transplant,” *National Post*, 12 September 2018, <https://nationalpost.com/feature/canadas-first-face-transplant>. [↑](#endnote-ref-76)
79. Margherita De Bac, “Transplanted Face Rejected: ‘Now We Need a New Donor,’” *Corriere Della Sera*, 25 September 2018, <https://www.corriere.it/english/18_settembre_25/transplanted-face-rejected-now-we-need-new-donor-0916a0ea-c0d2-11e8-8c2f-234b69fe8a3d.shtml>. [↑](#endnote-ref-77)
80. Ducharme, “Meet the First African-American Face Transplant Recipient.” [↑](#endnote-ref-78)
81. Michael Case, “New Hampshire Woman Becomes First Person to Get Second Face Transplant in U.S.,” *Time*, 7 August 2020, [https://time.com/5877653/carmen-blandin-tarleton-second-face-transplant/.](https://time.com/5877653/carmen-blandin-tarleton-second-face-transplant/#:~:text=New%20Hampshire%20Woman%20Becomes%20First%20Person%20to%20Get%20Second%20Face%20Transplant%20in%20U.S.&text=Carmen%20Blandin%20Tarleton%2C%20whose%20face,six%20years%20after%20the%20operation.) [↑](#endnote-ref-79)
